# Changing of mechanical property and bearing capacity of strongly chlorine saline soil under freeze-thaw cycles

**DOI:** 10.1038/s41598-024-56822-8

**Published:** 2024-03-14

**Authors:** Shijun Ding, Shaomin Li, Sen Kong, Qiuyang Li, Taohui Yang, Zhibao Nie, Gaowen Zhao

**Affiliations:** 1 School of Highway, Chang’an University, Xi’an, 710064 China; 2 Key Laboratory for Special Area Highway Engineering, Ministry of Education, Chang’an University, Xi’an, China; 3 China Electric Power Research Institute, Beijing, 100085 China; 4 State Grid Qinghai Electric Power Company, Xi’ning, 810001 China

**Keywords:** Strongly chlorine saline soil, Freeze-thaw cycles, Mechanical property, Subsoil-bearing capacity, Mechanisms, Civil engineering, Energy grids and networks

## Abstract

Freeze-thaw cycles and compactness are two critical factors that significantly affect the engineering properties and safety of building foundations, especially in seasonally frozen regions. This paper investigated the effects of freeze-thaw cycles on the shear strength of naturally strongly chlorine saline soil with the compactness of 85%, 90% and 95%. Three soil samples with different compactness were made. Size and mass changes were measured and recorded during freeze-thaw cycles. Shear strength under different vertical pressures was determined by direct shear tests, and the cohesion and friction angle were measured and discussed. Microstructure characteristic changes of saline soil samples were observed using scanning electron microscopy under different freeze-thaw cycles. Furthermore, numerical software was used to calculate the subsoil-bearing capacity and settlement of the electric tower foundation in the Qarhan Salt Lake region under different freeze-thaw cycles. Results show that the low-density soil shows thaw settlement deformation, but the high-density soil shows frost-heaving deformation with the increase in freeze-thaw cycles. The shear strength of the soil samples first increases and then decreases with the increase in freeze-thaw cycles. After 30 freeze-thaw cycles, the friction angle of soil samples is 28.3%, 29.2% and 29.6% lower than the soil samples without freeze-thaw cycle, the cohesion of soil samples is 71.4%, 60.1% and 54.4% lower than the samples without freeze-thaw cycle, and the cohesion and friction angle of soil samples with different compactness are close to each other. Microstructural changes indicate that the freeze-thaw cycle leads to the breakage of coarse particles and the aggregation of fine particles. Correspondingly, the structure type of soil changes from a granular stacked structure to a cemented-aggregated system. Besides, the quality loss of soil samples is at about 2% during the freeze-thaw cycles. Results suggest that there may be an optimal compactness between 90 and 95%, on the premise of meeting the design requirements and economic benefits. This study can provide theoretical guidance for foundation engineering constructions in seasonally frozen regions.

## Introduction

Natural saline soil is always located in saline or salt-lake regions, and being regarded as an inferior construction material due to its poor and complicated engineering properties. Saline or salt-lake regions always have obvious elevation variation and great climate change varying from subtropical to cold temperate, with temperatures ranging from − 33 to 35 °C. When the temperature is low, both salt and ice would crystallize together in saline soils, resulting in frost heave and salt expansion^1^. Thus, large temperature variations would cause significant and complex physical and chemical changes in saline soil, which has a great impact on engineering properties including volumetric behavior, shear strength and bearing capacity.

It is clear that freeze-thaw change is an important factor that induces soil structure changes in seasonally frozen regions. Wang et al.^2,3^ believed that the freeze-thaw cycles changed the pore structure of soil, destroyed the connection and arrangement of original particles, and then degraded the engineering properties of soil. Specifically, the freeze-thaw cycles promoted the increase in macropores and the development of microcracks. Han^4^ and Tao^5^ found that the freeze-thaw cycles not only caused a decrease in small pores and an increase in large pores, but also led to the breakage and aggregation of mineral particles. Shen et al.^6^ studied the effect of compaction on the saline soil under freeze-thaw conditions and found that the freeze-thaw cycles had a bidirectional effect on different-density soil, which increased the void ratio in dense soil and decreased the void ratio in loose soil. With the increase in freeze-thaw cycles, the void ratio gradually approached a stable value^7^. Moreover, the freeze-thaw cycles caused compression deformation on soil with a low compaction degree and uplift deformation on soil with a high compaction degree. After repeated freeze-thaw cycles, a critical compactness level was reached and the deformation of soil became stable. Research^8,9^ on the soil microstructure mainly focused on pores and particles, and believed that using SEM to observe changes in soil structure would help to understand the evolution of the soil microstructure under freeze-thaw cycles.

Some physical properties of soil including void ratio, density and permeability had been extensively studied in the early freeze-thaw cycles^10–12^. Wang^13^, Shah^14^ and Zhang^15^ studied the salt expansion of undisturbed saline soil under freeze-thaw cycles, and found that the pore water content in undisturbed saline soil affected the frost-heaving sensitivity of soil, which was related to the type and content of salts. Zhang^16^ had investigated the mechanisms and characteristics of salt expansion in saline soil, and found that the rate of salt expansion decreased with the increase in the compactness of soil, but increased with the increase in salt content. Other scholars^17–19^ had carried out relevant research on the shear strength of sulfate saline soil under the effect of freeze-thaw cycles, mainly using laboratory tests, with only 10 ~ 15 freeze-thaw cycles, and found that the cohesion and friction angle gradually decreased when the number of freeze-thaw cycles was less than 6. Due to the variety in the soil samples and testing conditions, the conclusions of the previous studies are not always consistent. For example, some scholars believed that the cohesion and friction angle of the soil decreased with the increase of freeze-thaw cycles and salt contents. However, some scholars argued that with the increase in freeze-thaw cycles, especially at a lower temperature, the cohesion gradually increased, but the friction angle decreased. Xia^20^ conducted a laboratory test of the natural soil, and the results showed that cohesion and uniaxial compressive strength decreased as the volume and porosity of the soil increased after experiencing various freeze-thaw cycles. Cheng^21^ studied the effects of the salt content on the strength and deformation characteristics to establish a function through triaxial compressive tests, which could interconvert the concentration of the sodium sulfate solution and the shear strength in the silty sand.

Types of salt in saline soil are the presently studied hotspots. Previous research^22^ showed that during the process of cooling and salt transferring, sulfate saline soil could combine with 7 or 10 water molecules to form dehydrated sodium sulfate (mirabilite) or sodium heptahydrate sulfate. This could cause the volume to increase by more than 200%, resulting in salt heaving in the soil. In terms of chlorine saline soil, many researchers^23–26^ measured the strength of the treated and untreated saline soil under short-term freeze-thaw cycles, and found that the treated saline soil gradually decreased, but the strength of untreated saline soil first increased and then decreased.

Previous studies well studied and summarized the changing of saline soil properties subjected to freeze-thaw cycles. However, most of the existing studies are aimed at conventional saline soil with relatively low salt content, and a large number of studies are based on artificially prepared saline soil, which is quite different from the saline soil in actual engineering, and hence cannot fully reflect the engineering characteristics of saline soil in the actual service process. Moreover, the research results of the shear strength characteristics of saline soils were mostly obtained under short-term freeze-thaw cycles, and the maximum number of freeze-thaw cycles was less than 10^27^. In the present study, nature strongly chlorine saline soil from the Qarhan Salt Lake region was selected to conduct this research. Qarhan Salt Lake region is located in northwest China and has special climatic and environmental conditions, with a large temperature difference between day and night in winter. According to the meteorological data, the minimum average temperature at night is about − 15 °C, and the highest average temperature during the day is about 20 °C. In this regard, during each winter, the natural saline soil experiences frequent freeze-thaw cycles, which have a great influence on its mechanical properties. Due to the special climatic conditions in the Qarhan Salt Lake region, the shear strength characteristics of the strongly chlorine saline soil under long-term freeze-thaw cycles remain to be studied. Therefore, to achieve the objective, direct shear tests were carried out on natural saline soil with different degrees of compaction (85%, 90% and 95%) under different short-term and long-term freeze-thaw cycles. Size and mass changes were measured and recorded during freeze-thaw cycles. Cohesion and friction angle were determined by direct shear tests. Microstructure characteristic changes of saline soil samples were observed using scanning electron microscopy (SEM) under different freeze-thaw cycles. Moreover, numerical software was used to calculate the subsoil-bearing capacity and settlement of the tower foundation under different freeze-thaw cycles. The increasing incidence of temperature fluctuations in the Qarhan Salt Lake region poses a growing threat to the integrity of power transmission infrastructure, leading to the subsidence of transmission tower foundations. If not promptly detected and addressed, this could gravely jeopardize the secure and stable operation of the electrical grid. To ensure the safe and stable functioning of existing power transmission lines in the Salt Lake region and the smooth construction of future projects, research into the adverse mechanisms affecting strongly saline soil foundations under freeze-thaw cycles induced by temperature differentials is being conducted. The research is instrumental in safeguarding the long-term stability of the electrical network in the Salt Lake region and also provides valuable insights for the engineering design, construction, and maintenance of foundations in strongly saline soils or Salt Lake region.

## Experimental program

### Materials

**Figure 1:**
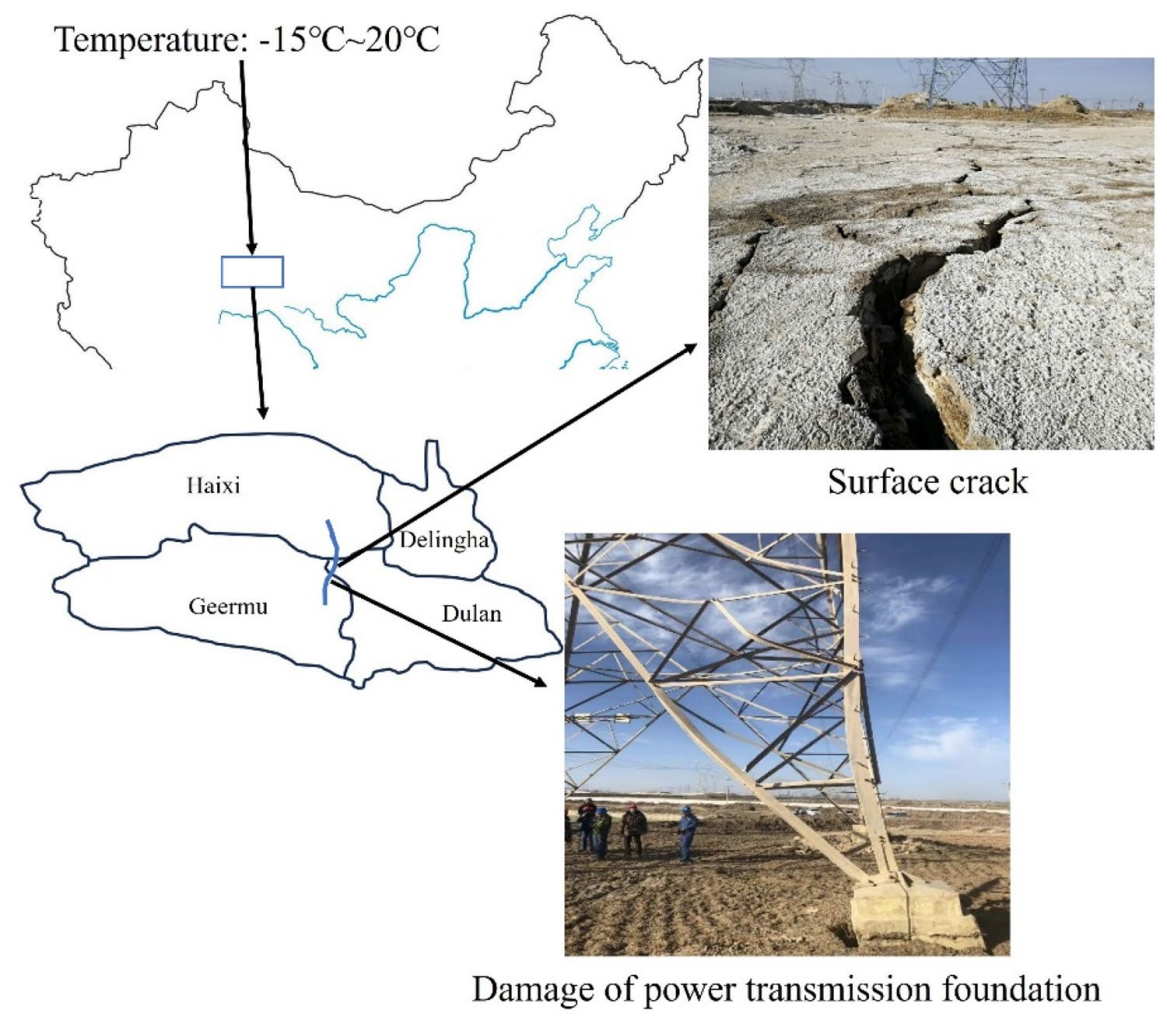
Location and engineering disaster of the Qarhan Salt Lake region.

Natural saline soil was collected from the damaged foundation field site of an electric power transmission in the Qarhan Salt Lake region (as shown in Fig. 1). The region is located in a place having a special climatic and environmental condition, which is harmful to engineering construction, such as surface cracks and electric tower deformation (Fig. 1). In the Qarhan Salt Lake region, the annual average rainfall is low, but the annual average evaporation is high, resulting in a high salt content in the lake water. Because the dry climate, a large amount of lake water evaporates and concentrates, leading to the deposition of salts and the formation of exposed land surfaces. The deposits consist of soil-cemented clastic layers. Therefore, the strongly saline soil was taken from below the foundation of the power transmission and transformation damage, about 3 m below the ground surface. Before determining the basic physical properties of saline soil, it is usually necessary to carry out desalination treatment on the saline soil. However, firstly, the natural saline soil used in the paper has a high salt content, and the salt plays a role in the soil particles. Secondly, in combination with the actual situation, pure water is added during laboratory experiments without adding brine, because brine will greatly change the original properties of the soil samples. Finally, the high salt content makes it difficult to determine the distribution of particles smaller than 0.074 mm, so we only measured the distribution of particles larger than 0.075 mm. Therefore, we performed compaction tests, boundary moisture content tests, salt dissolution tests and grain distribution analysis tests on the saline soil collected in the field according to the standard for soil test method (GB/T 50123-2019). The basic physical parameters and soluble salt content of saline soil are shown in

**Figure 2:**
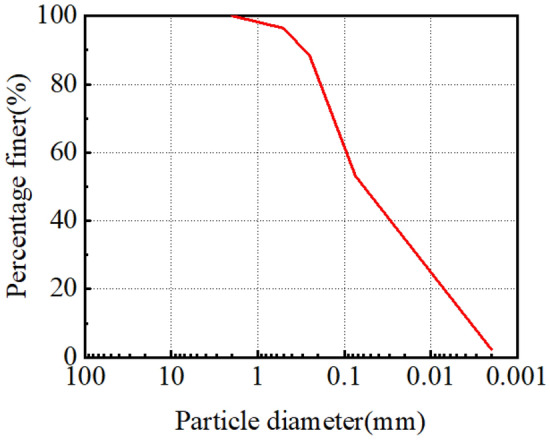
Grain-size distributions distribution curve of the test soil.

Tables 1 and 2. The grain size distribution is shown in Fig. 2. According to the technical code for building in saline soil regions (GB/T 50942-2014), the soil is characterized as strongly chlorine saline soil.

**Table 1:** Basic physical property parameters of soil.

Optimum moisture content / %	Max. dry density / g·cm^-3^	Liquid limit / %	Plastic limit / %
9.6	1.70	21.4	12.9

**Table 2:** Content of soluble salt.

Content of selected salts / mg·kg^-1^								Total salt content
Na^+^ + K^+^	Ca^2+^	Mg^2+^	CO_3_^2−^	HCO_3_^−^	Cl^-^	SO_4_^2−^	Cl^−^/SO_4_^2−^	/ %
145517	8885	6059	133	99	243,313	19,245	12.6	42.3%

### Sample preparation

**Figure 3:**
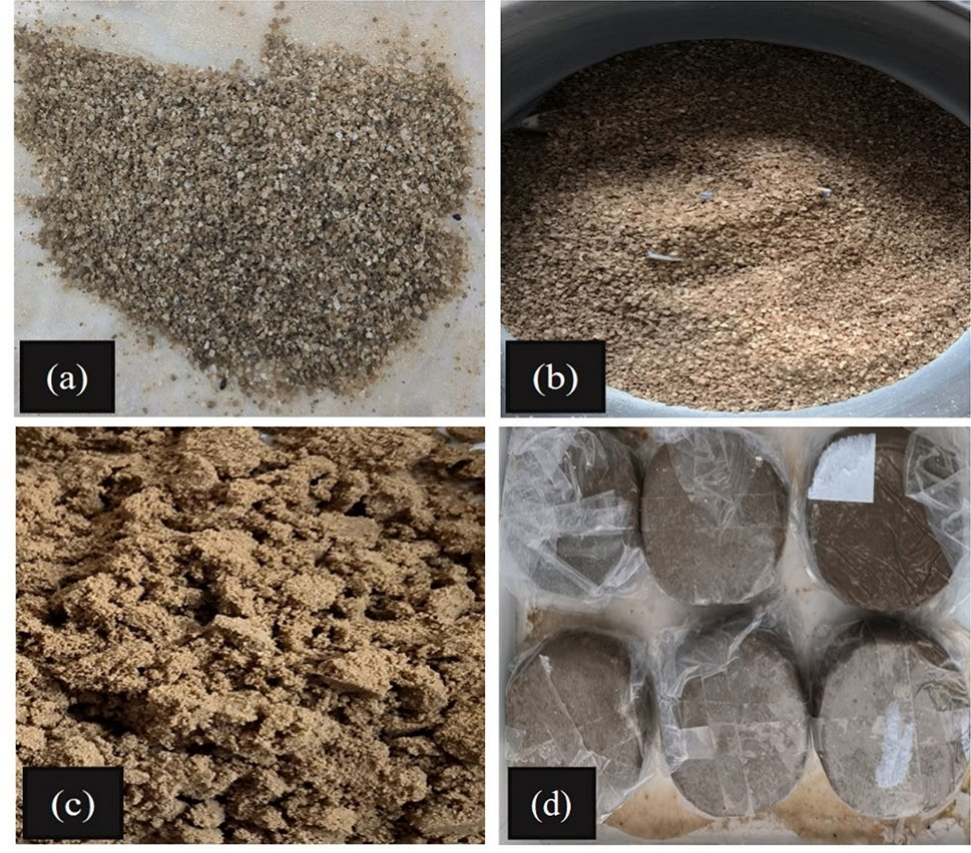
The process of sample preparation: (**a**) Drying natural saline soil; (**b**) Crushing natural saline soil; (**c**) humidifying natural saline soil; (**d**) Sample preparation and package.

According to the Qarhan Salt Lake region investigation, the annual maximum surface temperature is around 50 °C, so setting the drying temperature to 50 °C conforms to the actual working conditions. Secondly, a study found that the water content of different types of saline soil did not change significantly when dried at a temperature below 60 °C. However, when the drying temperature exceeded 60 °C, a significant loss of crystalline water from the salt began, leading to a noticeable change in water content. It was suggested that the water content of saline soil should be determined at a temperature below 60 °C, so we get the water content of soil samples at 50 °C. Finally, the drying time of soil samples in the oven is relatively long, and we have done some verification tests to ensure that the moisture content is basically the same everywhere. Therefore, we set the drying temperature to 50 °C and ensure the soil samples are dried. The preparation process of soil samples is shown in Fig. 3. Firstly, the collected natural saline soil was oven-dried at 50 °C, crushed, and passed through a 2 mm sieve. Secondly, freshwater was evenly sprayed on the sieved dry soil and thoroughly mixed until the optimum water content (9.6%) was reached. Thirdly, the mixed soil was sealed and let stand for 24 h to make the moisture distribution in the soil more uniform. Then, the soil samples were remoulded into cutting ring samples with 20 mm in height and 61.8 mm in diameter, using the one-step compaction moulding method. Finally, the cutting ring samples were wrapped hermetically in fresh-keeping film and placed in an incubator to prevent water loss. Considering the practical application in transmission line engineering, selecting 85% of maximum dry density, 90%, and 95% as the initial compaction degrees of the soil samples (hereafter referred to as C85, C90, and C95, respectively), and corresponding to dry densities of 1.45 g cm^−3^, 1.53 g·cm^−3^, and 1.62 g·cm^−3^, respectively.

### Freeze-thaw procedure

**Figure 4:**
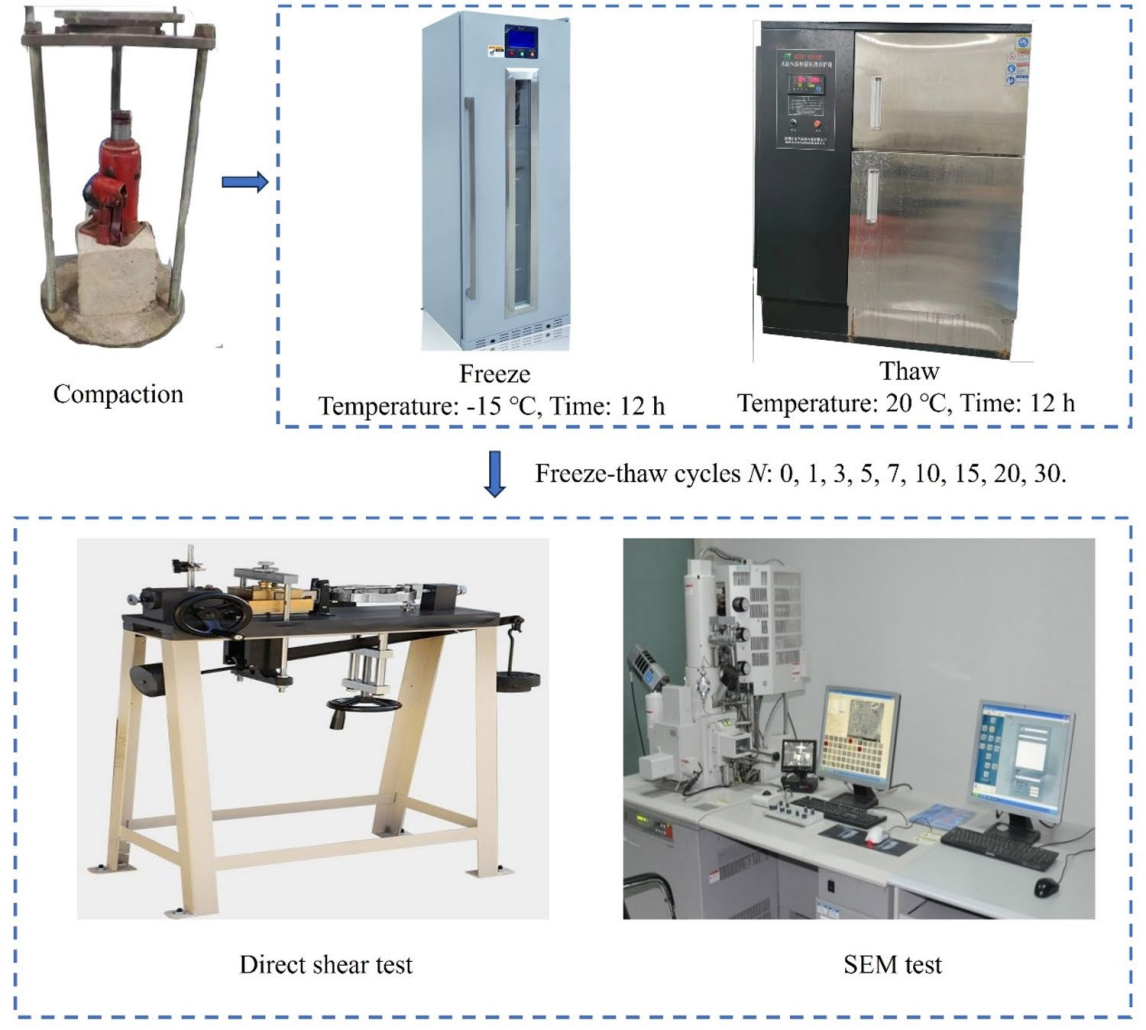
Flowchart of test scheme.

To simulate the freeze-thaw cycle process of the practical condition in the subsoil, 30 freeze-thaw cycles were performed for the soil samples on the incubator and refrigerator. The freezing temperature was set to − 15 °C and the melting temperature to 20 °C. The soil samples were frozen for 12 h, and thawed for 12 h, so a total of 24 h was needed for each freeze-thaw cycle. The number of freeze-thaw cycles N was set at 0, 1, 3, 5, 7, 10, 15, 20, and 30. The test flowchart is shown in Fig. 4.

### Test methods

#### Size and mass changes

Three soil samples with different compactness were selected, and the size and mass changes were measured and recorded during freeze-thaw cycles. After each freeze-thaw cycle was completed, the quality of soil sample was measured by an electronic scale, the sample height changes at five 5 locations on the surface of the soil samples were measured by a vernier calliper, and measured data were averaged to assess the volumetric and qualitative change of saline soil.

#### Direct shear tests

The direct shear tests were conducted, using the YYW-2 strain-controlled direct shear apparatus for all soil samples, to determine the shear strength of the soil samples under different vertical stresses. cohesion and the friction angle had been calculated. To conduct the direct shear test, the soil sample was placed in the direct shear apparatus. Constant vertical stress was applied to the sample, and initial normal stresses of 100, 200, 300, and 400 kPa at the shear plane were chosen. The soil is silt and has a high salt content, resulting in poor permeability. Therefore, according to the actual working conditions, the direct shear test is conducted using non-consolidated rapid shearing. The tests were conducted at a speed of 0.08 mm/min. During the direct shear test, the dial gauge reading is recorded every 15 s. The test ends when the dial gauge reading suddenly drops and remains unchanged. After the test, the shear stress is calculated based on the recorded data. Moreover, for the samples in the same load conditions, the soil cohesion (*c*) and internal friction angle (*φ*) were determined by using different shear stresses under the previously mentioned four initial normal stresses by following the Mohr–Coulomb criterion of failure.

#### Microscopic tests

**Figure 5:**
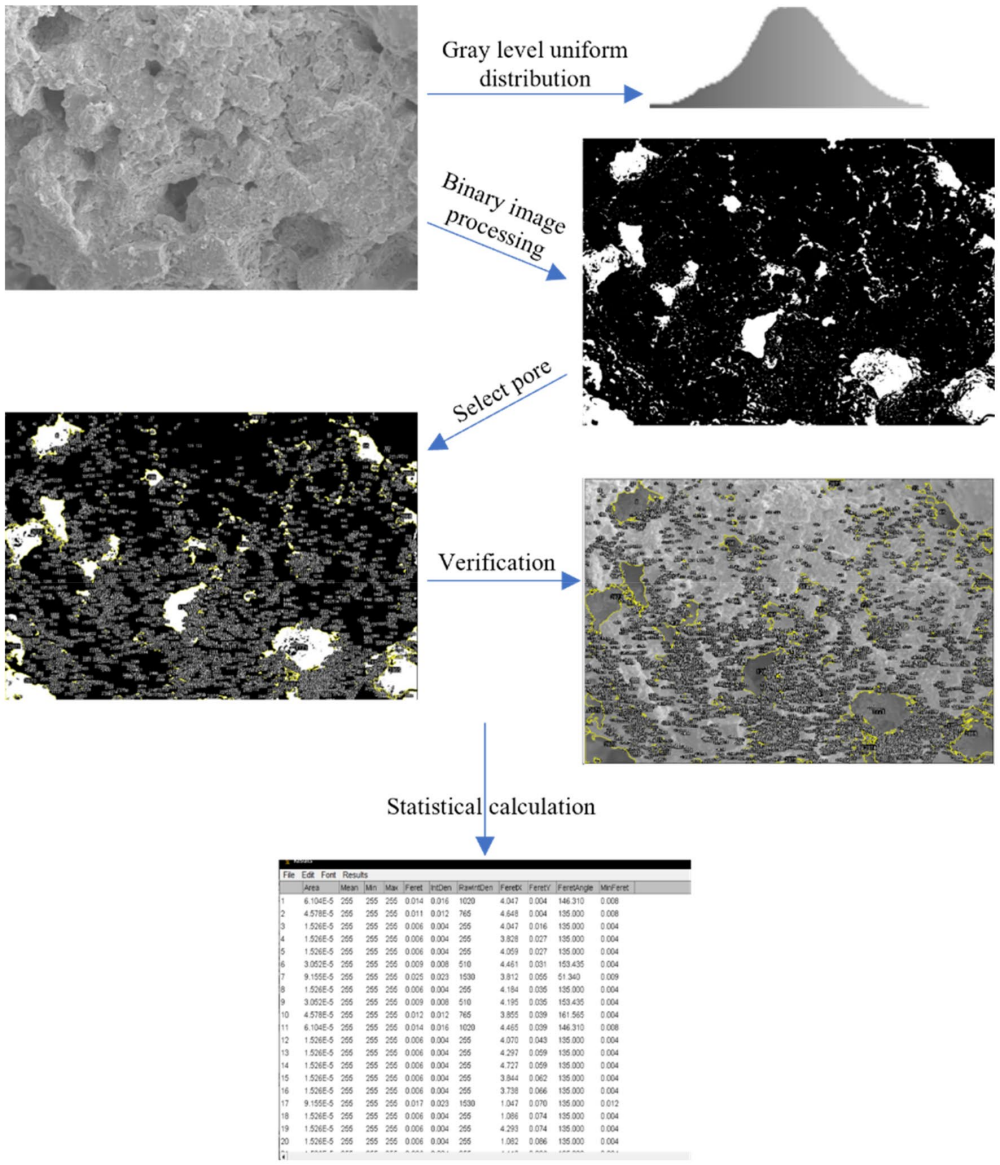
Quantitative analysis flowchart.

The microstructure characteristics of each compacted soil sample after different freeze-thaw cycles were observed by SEM (HITACHI0-S-4800). The soil sample was plated with a layer of gold film before observation, and the voltage was 10 kV during scanning. During microscopic testing, multiple different slices are taken from the center of the soil sample, each with a length and width of 10 mm and a height of 5 mm, and are dried at 50 °C. For soil samples under the same conditions, multiple slices and observation points are selected while scanning electron microscope observation, and select representative SEM images from them to ensure the accuracy of the results. The SEM images were obtained at 800 × and 2000 × magnification. To quantitatively analyze the effects of compactness and freeze-thaw action on the microstructure of strongly chlorine saline soil, the SEM images were digitized and various microstructural indicators were calculated using the image recognition and analysis software ImageJ. The specific process is shown in Fig. 5. Firstly, the images were subjected to grayscale processing, background elimination, and contrast enhancement. Secondly, to quantitatively analyze the changing characteristics of micro-pores, the images needed to be binarized, and an appropriate threshold was selected to generate a binary image. Finally, the pore size, dimension, area, etc. were set, and the pore structure was quantitatively analyzed.

### Computational procedure

#### Calculation process

**Figure 6:**
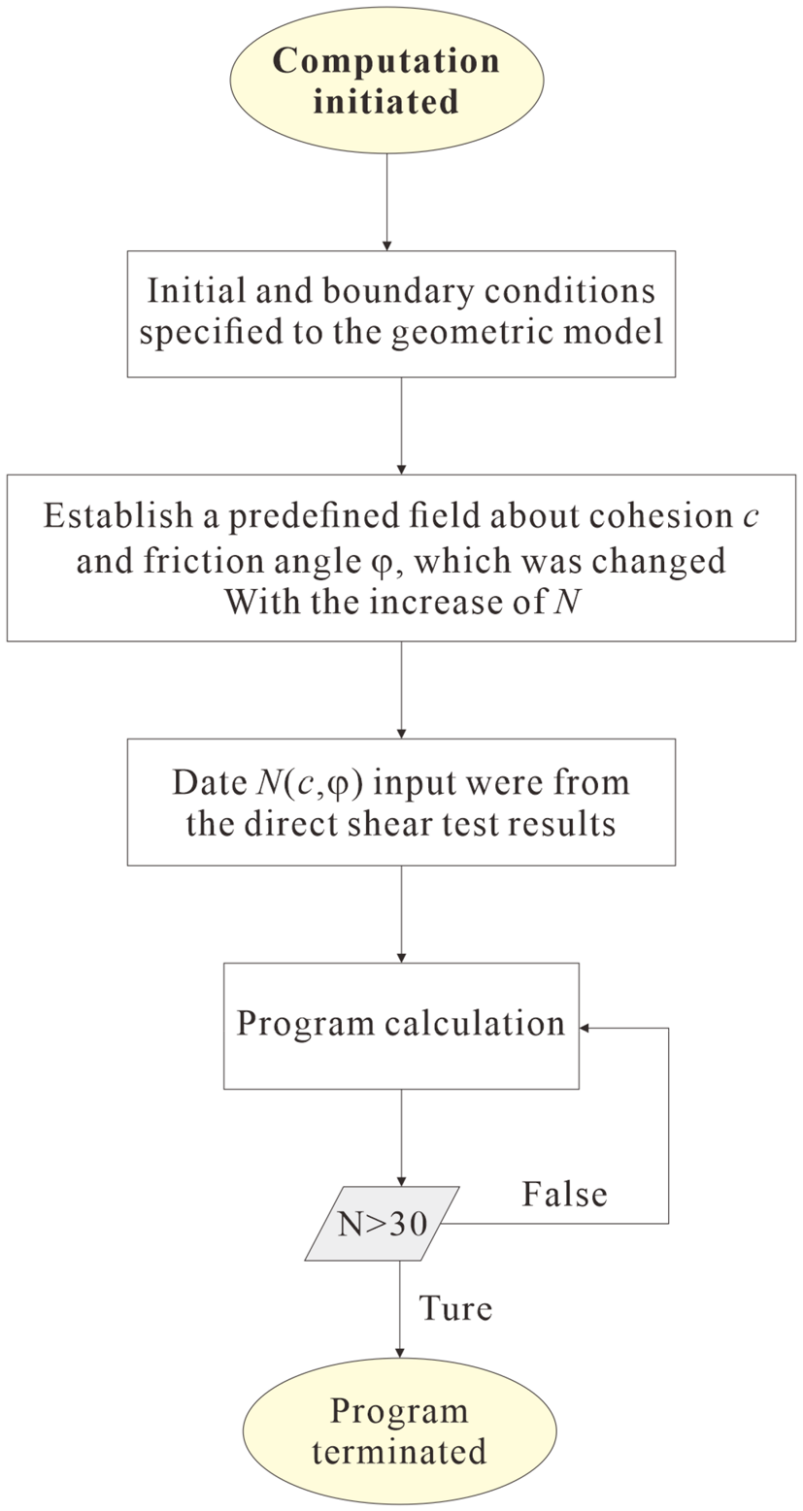
Flowchart of computational procedure.

The main computational procedure is shown in Fig. 6. Firstly, setting initial boundary conditions and balancing geo-stress. Secondly, establish a predefined field about cohesion and friction angle, which was changed with the increase of *N*. Then, the characteristic parameters for a numerical model were input according to the direct shear test results, and subsoil-bearing capacity and the settlement of foundation are calculated for 0 freeze-thaw cycle. Finally, Repeat the calculation until the 30 freeze-thaw cycles.

#### Finite element model (FEM)

In power transmission and transformation engineering, the bottom size of the inclined column foundation in the Qarhan Salt Lake region is 4.2 m × 4.2 m, the buried depth is h = 3 m, the superstructure exerts axial pressure on the foundation through bolts, and the design value of pressure is *N*_E_ = 1107 kN. Besides, the standard pressure value of the bottom surface of the foundation under different pressures is shown in Table 3.

**Table 3:** The foundation pressure under different axial loads.

The axial load / kN	Pressure / kPa
1107 (The design value *N*_E_)	62.8
–	8.5 (Overburden *p*)
1764	100
1107(*N*_E_) + 563(The deadweight of foundation G) + 8.5(*p*)	103.2

**Figure 7:**
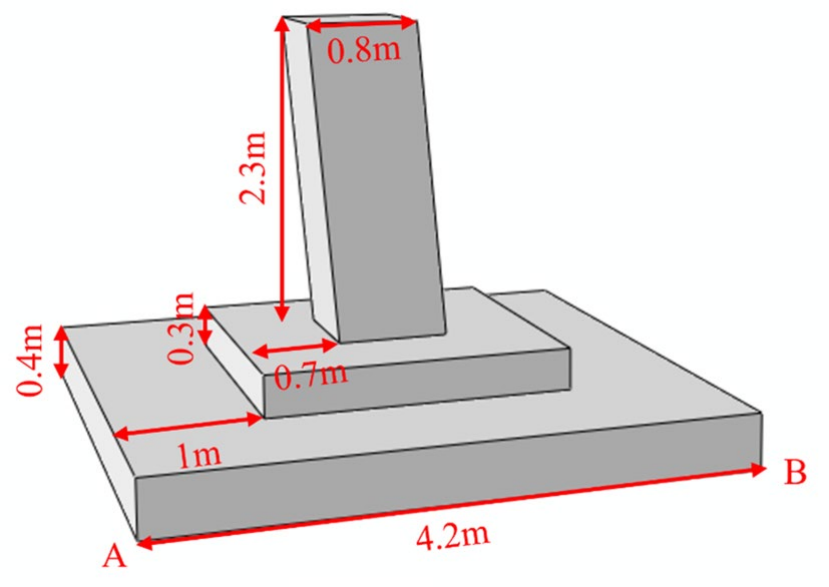
Geometric model and boundary conditions.

Based on the finite element method and ABAQUS 2018, the influence of freeze-thaw cycles on foundation soil was considered, and the additional stress of the foundation, foundation bearing capacity and the settlement of the tower base under vertical load. This paper analyzed the variational displacement of foundations under different loads and adopted multi-condition analysis by modifying the shear strength parameters of soil. Moreover, to calculate the subsoil bearing capacity, the foundation was loaded by applying axial load to the tower base model. The geometric model of the pole tower foundation, as illustrated in Fig. 7, corresponds to the actual working conditions. The material of the tower foundation was concrete that applied an elastic model, and the Morh-Coulomb model was applied for subsoil. The element type was C3D8, the contact relationship between the foundation and subsoil was established by the surface-to-surface function, the contact relationship was set as rigid contact, and the friction coefficient was 0.3. Material parameters are shown in Table 4.

**Table 4:** Material parameter.

Type	Subsoil	Foundation
Modulus of elasticity /MPa	10 (C85 and C90), 35 (C95)	20000
Poisson's ratio	0.38	0.2
unit weight / kN·m^-3^	17.6 (C85 and C90), 18.7(C95)	20

**Figure 8:**
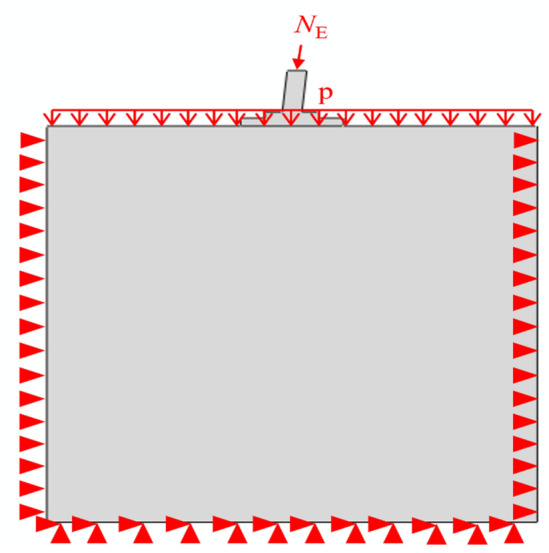
Boundary conditions.

The boundary conditions and loads are shown in Fig. 8. All nodes were constrained on the outer surface of the subsoil. At the same time, symmetric boundary conditions were imposed on the symmetric plane. A vertical load was distributed according to a uniform force applied on the top of the foundation soil to consider the influence of backfill. An axial load was also applied to the centre of the top surface of the foundation. The axial load was 1764 kN and increased step by step.

#### Analysis step

When the geostatic pressure was applied, only the geostatic pressure of the subsoil was read, but the initial stress of the foundation was not read. All boundary conditions were imposed in the initial analysis step, and all contact relations were specified. A geostatic analysis step is added after the initial analysis step, and there is only gravity load in the geostatic step so that the initial geostatic pressure could be well balanced well. After the geostatic analysis step, five static analysis steps were specified, and the loaded difference between two adjacent static analysis steps is 100 kN. Turn on the automatic time step function, and set the Initial time step as 0.01. The maximum time step was 0.5, and the minimum time step was 1E-5. The maximum number of steps was 1000.

## Results

### Appearance change

According to the change of sample appearance under different freeze-thaw cycles, representative sample photos are selected as shown in Table 5. After 1 freeze-thaw cycle, salt crystals gathered on the surface of soil samples. After 7 freeze-thaw cycles, many pores appeared on the surface of the soil samples. After 30 freeze-thaw cycles, the surface of the soil samples was uneven. The reason is that water and salt transfer caused by freeze-thaw led to the aggregation of salt and the formation of salt crystals, and the soil structure changes. With the increase in freeze-thaw cycles, the salt crystals gradually disappeare, but the soil structure could not be restored to its original state, which finally led to the uneven surface of the soil samples.

**Table 5:**
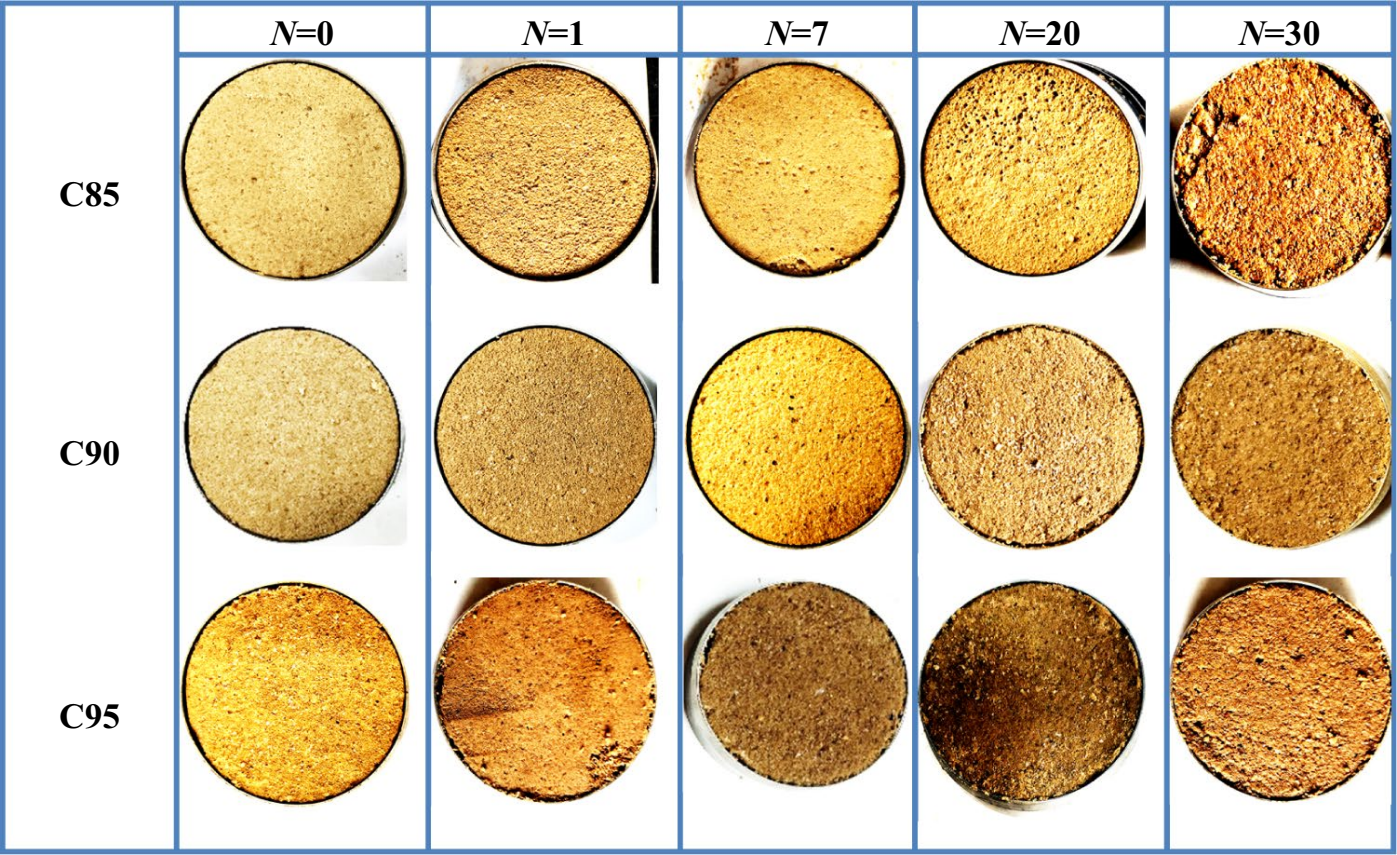
Photos of samples at various freeze-thaw cycles.

### SEM images

#### Qualitative analysis

**Figure 9:**
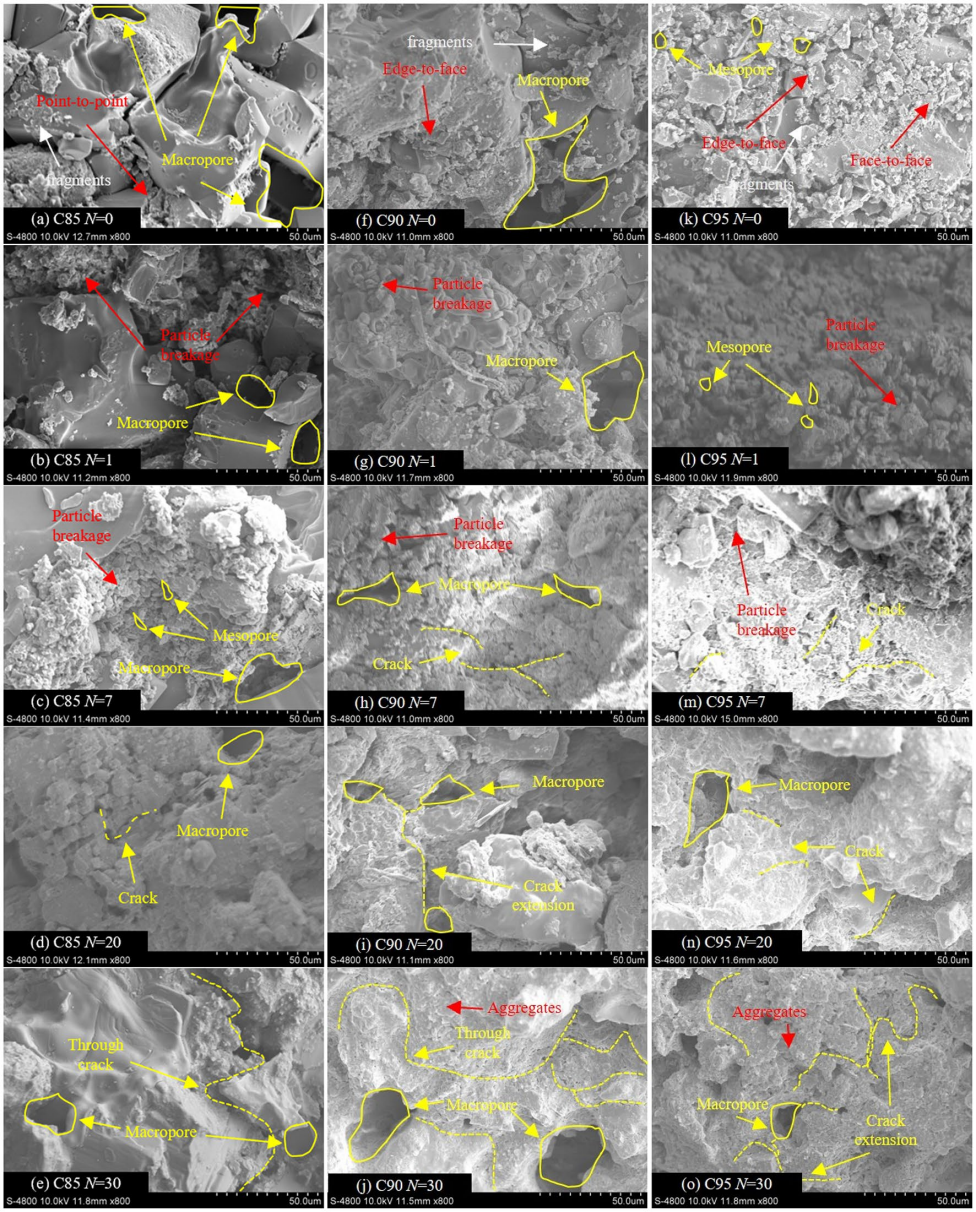
SEM images of soil samples after different freeze-thaw cycles (800 ×).

**Figure 10:**
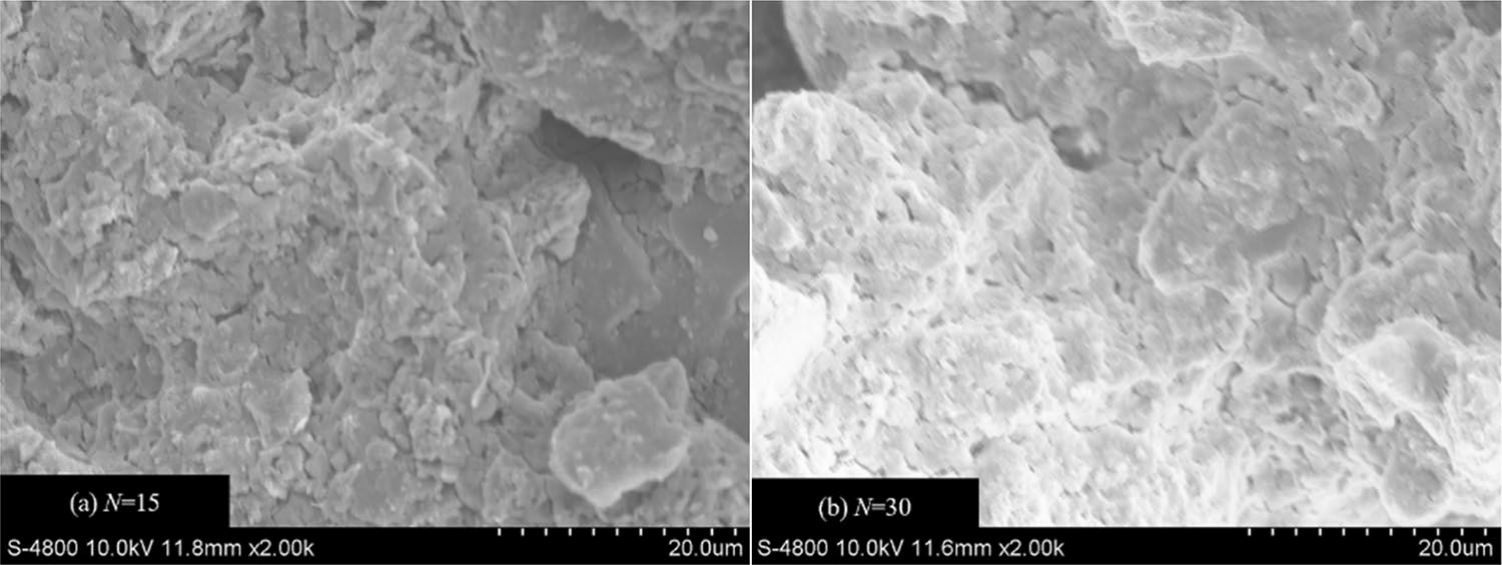
SEM images of soil samples after different freeze-thaw cycles (2000 ×).

The microstructure of the soil samples was qualitatively analyzed by SEM images at 800 × (Fig. 9) and 2000 × (Fig. 10) magnification. First, the images of the three unfrozen soil samples in Fig. 9a, f and k show that with the increase in compaction, the contact area between particles in the soil samples increases significantly, and the contact relationship gradually changes from point-to-point contact to edge-to-face contact. Moreover, the number of macropores is decreasing, and the soil structure gradually becomes denser^28^. In the C85 soil sample, the macropores are visible, and the soil particles are mainly connected by point-to-point contact. The C90 soil sample has a relatively loose structure with well-developed pores. On the contrary, in the C95 soil sample, the number of macropores is decreasing, the mesopores are visible, and the connection relationship between particles is mainly edge-to-face contact. Besides, due to the high salt content, a large number of irregular-shaped salt and soil fragments are loosely attached to the structural surface.

After the first freeze-thaw cycle, the particle morphology, pore distribution, and structural surface characteristics of the soil samples all change significantly. Figure 9b, g and l shows that the larger soil particles are broken, and the size of pores decreases. During the short-term freeze-thaw cycles (< 7), the number of macropores in the C85 soil sample is significantly reduced and appears as mesopores. In the C90 soil samples, the originally developed macropores change to mesopores and gradually connect into cracks, while in the structure of the C95 soil samples, the originally developed mesopores gradually connect into cracks.

**Figure 11:**
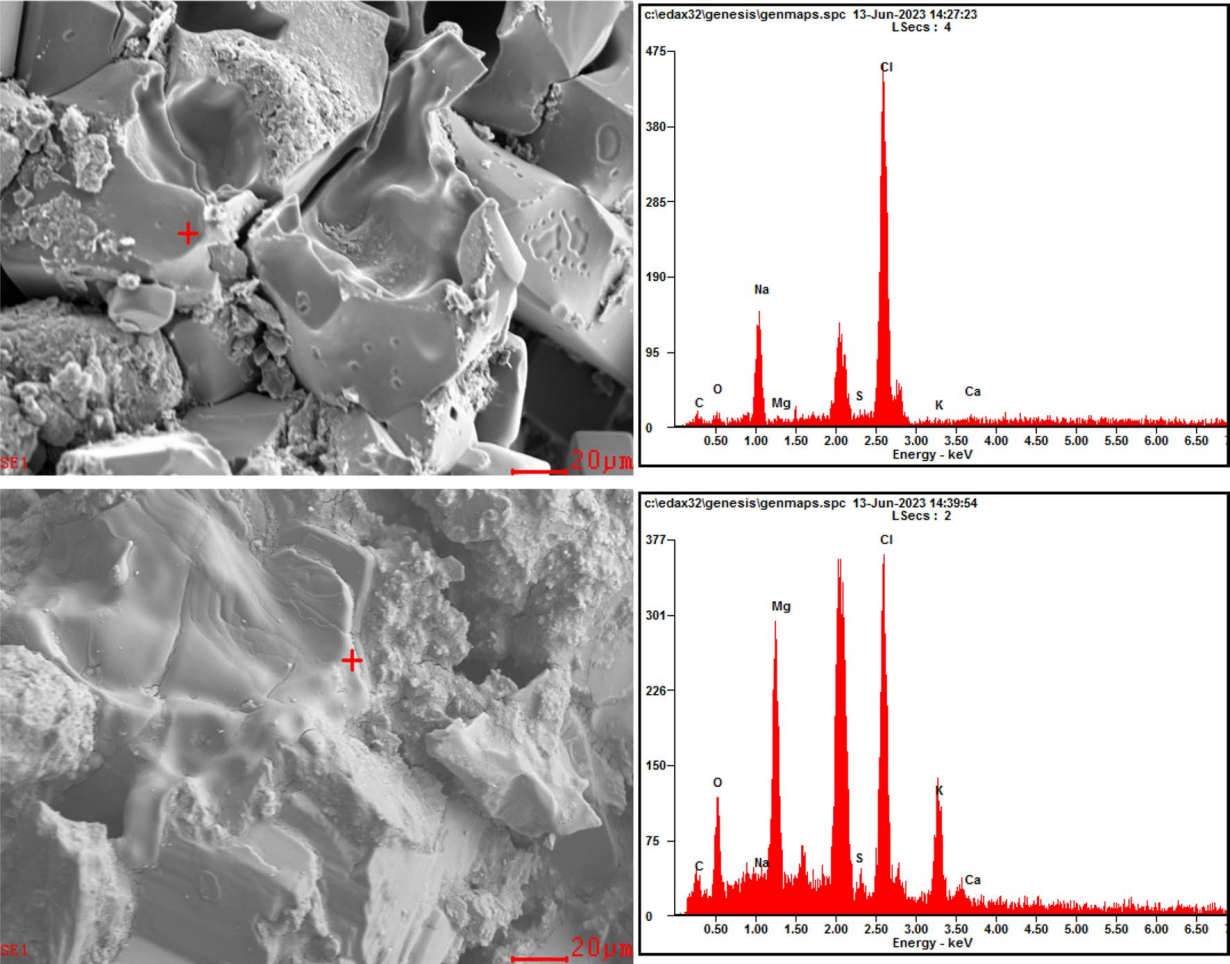
Analysis of elemental composition in strongly saline Soil subjected to freeze thaw cycles.

After further freeze-thaw cycles, the cracks continue to extend in the soil, causing some large particles to be further broken into a large number of fine particles (Fig. 9d, i and n), and the soil samples present a stacked structure (Fig. 10a). As shown in Fig. 9e, j and o, when the freeze-thaw cycles reach 30 times, the most significant change is that the macropores in the soil are connected by cracks, and the broken particles gradually gather together and form large aggregates under the cementation. At this time, the microstructure characteristics of the three soil samples tend to be similar, and the connection relationship between aggregates gradually becomes point-to-face and point-to-point contraction (Fig. 10b). Simultaneously, the skeleton particles form a stable aggregated structure under the attachment of cementing substances and crystalline salts. This process occurs during the long-term freeze-thaw cycles (> 10). Besides, according to the results of EDS analysis (Fig. 11), the soil and salt aggregates contain chlorine, sodium and magnesium, which are no different from those contained in soil samples without the freeze-thaw cycle, so only physical and crystallization changes occur during the formation of aggregates.

#### Quantitative analysis

**Figure 12:**
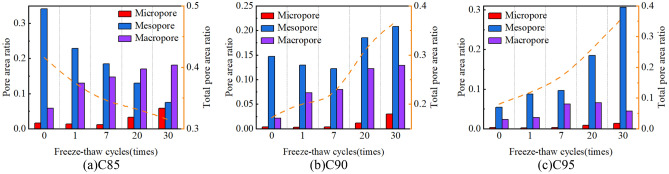
changes in the pore area ratio of the soil samples under different freeze thaw cycles.

With the increase in freeze-thaw cycles, the changes in the pore area ratio of the soil samples are illustrated in Fig. 12. The area of micropores ranges from 0 to 0.00785 μm^2^, the area of mesopores extends from 0.00785 to 0.785 μm^2^, and the area of macropores exceeds 0.785 μm^2^. The changes in pore area within the soil samples under different conditions are primarily in the mesopores and macropores. This is mainly manifested in the following three points: (1) The mesopore area ratio of C85 decreases with the increase in cycle numbers, but the macropore area ratio gradually increases. (2) The macropore area ratio of C90 gradually increases. (3) The mesopore area ratio of C95 gradually increases. Besides, the total pore area ratio of C85 decreases with the increase in cycles, but the total pore area ratio of C90 and C95 increases with the increase in cycles.

**Figure 13:**
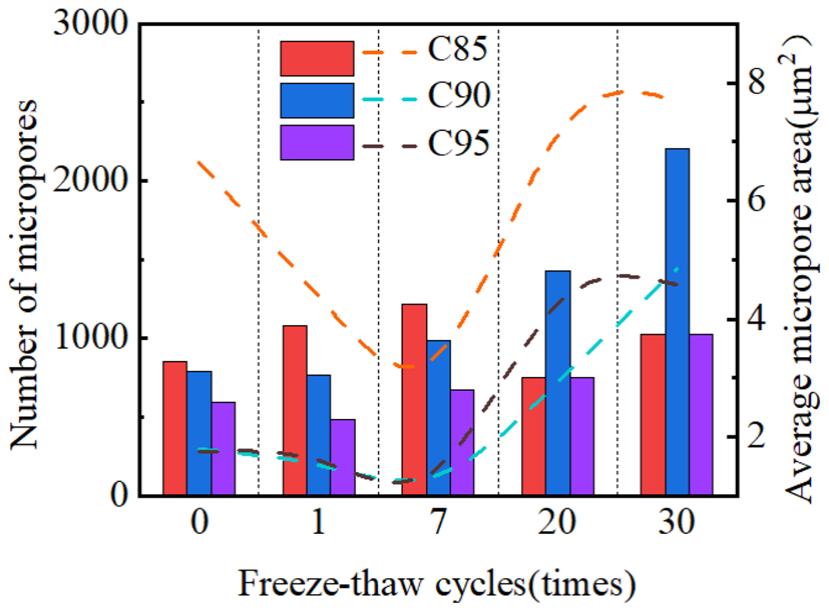
Number and average area of micropores under different freeze-thaw cycles.

As the number of freeze-thaw cycles increases, the changes in the number of micropores in the soil samples and the average area of the micropores are shown in Fig. 13. With the increase in cycles, the number of micropores in C85 and C95 changes little, fluctuating around 1000, but the number of micropores in C90 increases significantly. In addition, in the short-term freeze-thaw cycles, the average area of micropores in samples under different conditions all decrease. Conversely, in the long-term freeze-thaw cycles, the average area of micropores in samples under different conditions all increase.

### Sample quality

**Figure 14:**
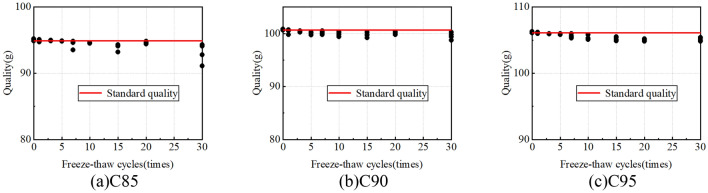
Sample quality under different freeze-thaw cycles.

The standard qualities for C85, C90, and C95 are 94.9, 100.6, and 106.1 g, respectively, representing the initial qualities of soil samples at varying degrees of compaction. When the soil samples complete the predetermined freeze-thaw cycles, peel off the plastic wrap wrapped around the sample weigh the sample's quality, and strictly prevent part of the soil from falling off the samples during this process. The sample quality under different freeze-thaw cycles is shown in Fig. 14. As can be seen from the figure, the quality loss of the samples is strictly controlled at about 2%. Therefore, it can ensure that the moisture content of the samples remains unchanged during the freeze-thaw cycle and ensure the accuracy of the test results.

### Volumetric changing behavior

**Figure 15:**
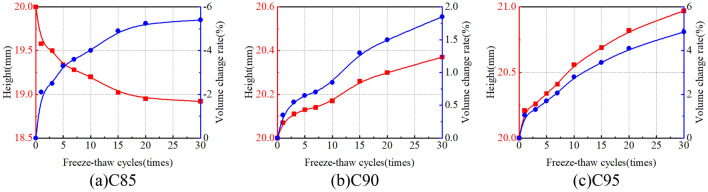
Variation curves of volumetric change under freeze-thaw cycles.

The impact of freeze-thaw cycles on the volume of soil samples is illustrated in Fig. 15. It is apparent from Fig. 15 that the volume of C85 decreases during freeze-thaw cycles, and the volumetric change rate is − 5.40% after 30 freeze-thaw cycles. On the contrary, the volumetric of C90 and C95 increase during freeze-thaw cycles, and the volumetric change rates are 1.85% and 4.85% respectively. This shows that compaction has an important effect on the volume of soil samples. Under the effect of freeze-thaw cycles, the low-density soil structure gradually becomes denser, showing thaw settlement deformation, but in the high-density soil sample, the soil structure becomes loose, showing frost-heaving deformation^29^.

### Shear strength parameter

#### Friction angle

**Figure 16:**
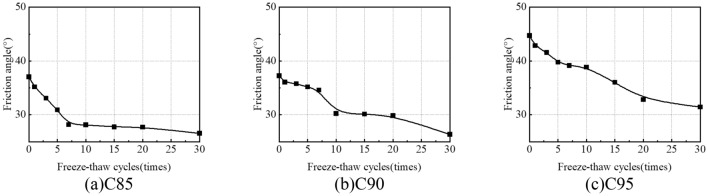
Variation curves of internal friction angle under freeze-thaw cycles.

Figure 16 shows the variation of the friction angle of the samples with the freeze-thaw cycles. The friction angle decreases with the increase of freeze-thaw cycles. After 30 freeze-thaw cycles, the friction angles of the soil samples are 26.65° (C85), 26.37° (C90) and 31.47° (C95), which are 28.3%, 29.2% and 29.6% lower than the soil samples without freeze-thaw cycles. It is because a part of large-size soil is decomposed into small-size soil leading to the pore structure in soil changes, and the migration of water in soil also changes the internal structure of soil and redistributes soil particles during the freeze-thaw cycles, resulting in decreasing biting force between soil particles. Finally, the sliding frictional force between soil particles decreases. Therefore, the friction angle decreases with the increase of freeze-thaw cycles.

The friction angle of C85 decreases rapidly during the short-term freeze-thaw cycles (< 7), as shown in Fig. 16a, and stabilizes at about 27 during the long-term freeze-thaw cycles (> 10). On the contrary, the friction angle of C90 and C95 decreases slowly during the short-term freeze-thaw cycles (< 7), as shown in Fig. 16b, c, and decreases rapidly during the long-term freeze-thaw cycles (> 10). Compared with C85, the friction angle of C90 and C95 decreases more slowly during the short-term freeze-thaw cycles (< 7), because the degree of compaction affects a cementing capacity between soil particles, which is beneficial to improve the stability of soil structure because the degree of compaction affects the cementing capacity between soil particles, which is beneficial to improve the stability of soil structure, thus repressing the friction angle decreases. However, during the long freeze-thaw cycle (> 10), soluble salts are crystallized among the soil particles, which increases the volume of the soil and results in increasing particle voids, particle rearrangement and decreased cementing capacity, and so the friction angle of C85, C90 and C95 will eventually stabilize around 25–30°.

#### Cohesion

**Figure 17:**
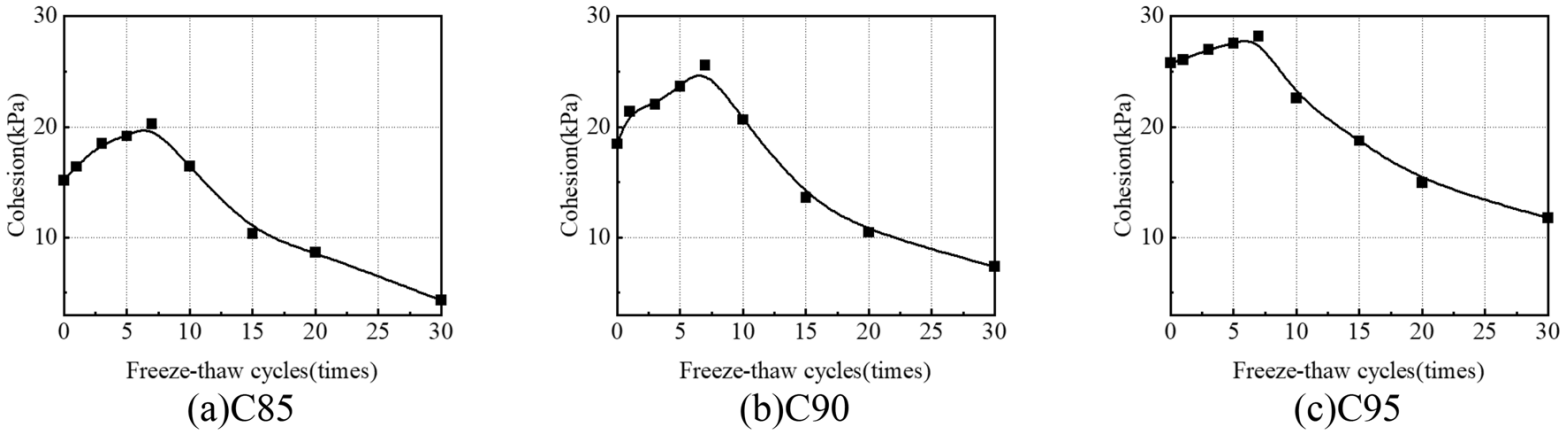
Variation curves of cohesion under freeze-thaw cycles.

Figure 17 shows the variation in the cohesion of the soil samples with freeze-thaw cycles. The cohesion first increases and then decreases with the increasing freeze-thaw cycles. After 30 freeze-thaw cycles, the cohesion of the soil samples is 4.34 kPa (C85), 7.93 kPa (C90) and 11.76 kPa (C95) respectively, which are 71.4%, 60.1% and 54.4% lower than the soil samples without freeze-thaw cycles (The experimental results are the same as Jia research^30^). The cohesion first increases because small-size soil particles and salt crystals accumulate into large soil aggregates that contain soil and salt during the short-term freeze-thaw cycles (< 7), which enhances the bond strength between particles. Besides, water freezing leads to soil expansion, which has a compaction effect on soil structure, improving the cohesion of soil. The reason for the decrease in cohesion is that during the long-term freeze-thaw cycles (> 10), the decrease in cementing ability between soil particles leads to the formation and break of soil aggregates, which changes the internal structure of soil and forms a macroporous structure, which decreases the bond strength between particles, so the cohesion of soil decreases with the increase of freeze-thaw cycles.

### Stress–strain curves

**Figure 18:**
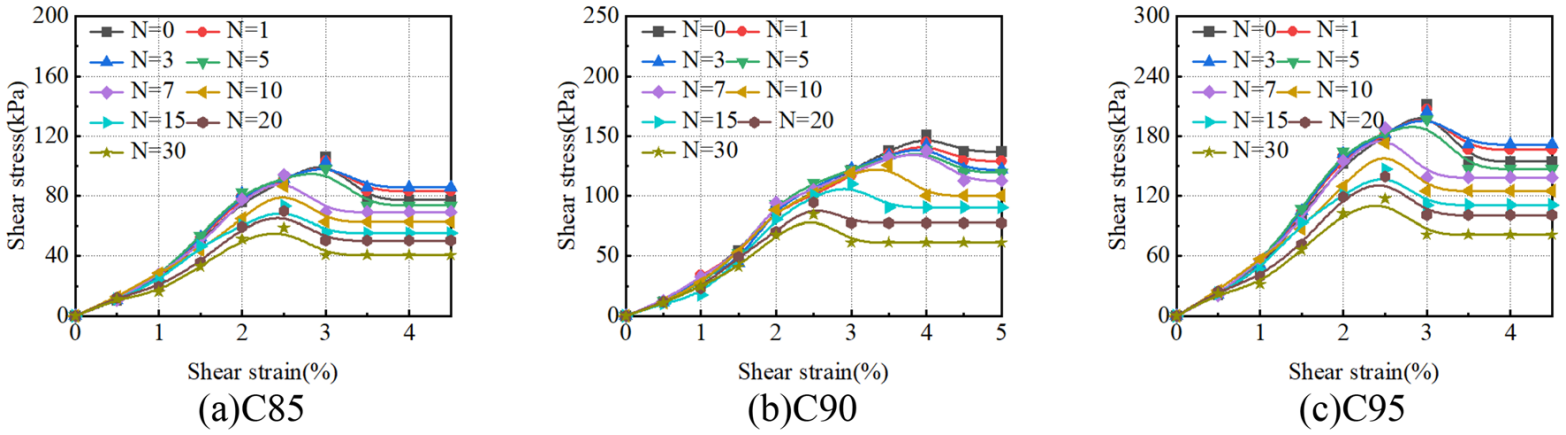
Stress–strain curves under freeze-thaw cycles.

Direct shear tests were conducted at different freeze-thaw times for th soil samples. According to the actual working conditions of the tower foundation in the Qarhan Salt Lake region, take C85 as an example, the stress–strain curves for the soil samples at initial normal stresses of 100 kPa were presented respectively in Fig. 18.

As shown in Fig. 18, the stress–strain curves show a strain-softening behaviour for the soil samples. Before the shear stress reaches the peak, it gradually increases with the increase of shear strain, and the increase of rate gradually slows down, finally tending to be stable. After the shear stress reaches the peak, it first decreases to a certain value and then does not increase with the increase of shear strain. The maximum shear stress on the stress–strain curves coincides during the short-term freeze-thaw cycles (< 7) and is a big difference during the long-term freeze-thaw cycles (> 10). The main reason for the phenomenon is that the soil samples undergo three stages under an axial load of 100 kPa during the direct shear test: (1) Within the elastic proportional limit, the shear strain of the soil samples is less than 2%, which is the process of soil compaction. During short-term freeze-thaw cycles (< 7), for the same Compactness, the soil samples with different freeze-thaw cycles have the same type of pores, hence the curves almost coincide. During long-term freeze-thaw cycles (> 10), it is the process of soil crack densification, but the crack morphology of soil samples with different freeze-thaw cycles is different, hence the curves do not coincide. (2) With the increase of shear stress, soil particles undergo sliding motion, mainly relying on the soil structure to provide strength. (3) After the peak, the soil structure is destroyed and reduced to a balance of frictional forces between the upper and lower shear surfaces, at which point the shear stress is residual strength. During long-term freeze-thaw cycles (> 10), the forms of structural damage of aggregates are different with different freeze-thaw cycles. Therefore, the freeze-thaw effect after the shear stress peak becomes more and more pronounced. It shows that the mechanical properties of the soil sample are unstable after many freeze-thaw cycles.

### Shear modulus

The shear modulus in the shearing process is constantly changing. To better and more accurately investigate the behaviour of soil samples, the shear modulus comes from the stress–strain curve (Fig. 18) for the initial normal stresses of 100 kPa. The shear modulus as a parameter to indicate the rigidity of the samples is measured by the slope of a straight line from the origin to the point equivalent to 2% of the strain in the stress–strain curve.

**Figure 19:**
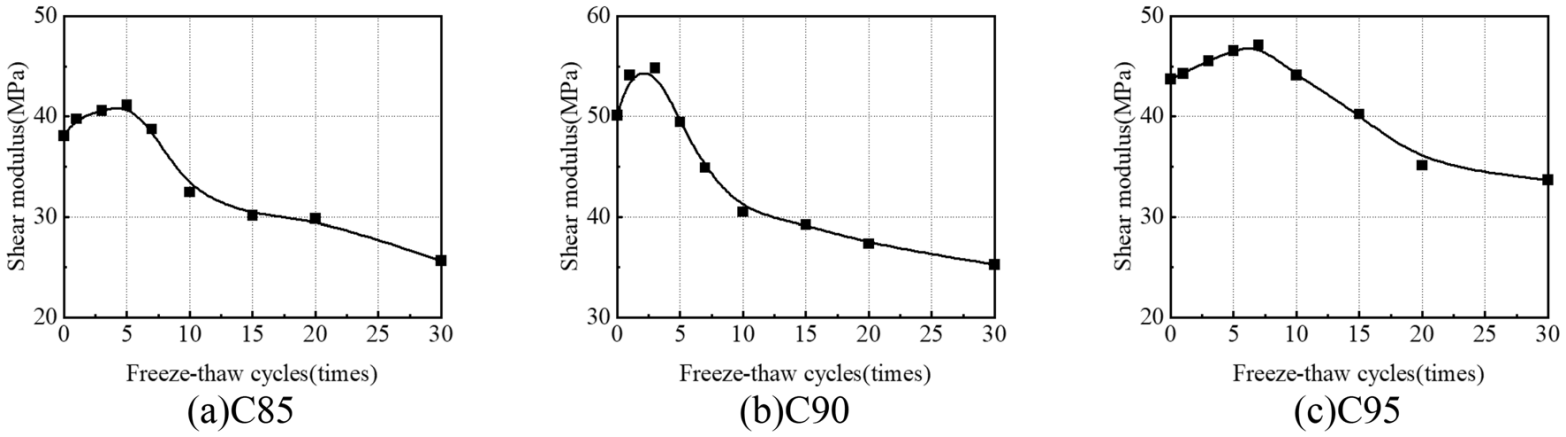
Variation curves of Shear modulus under freeze-thaw cycles.

The shear modulus is as shown in Fig. 19. Enhancing the compaction of soil samples is conducive to bolstering their capacity to resist shear deformation. The shear modulus first increases and then decreases with the increase in freeze-thaw cycles. After 30 freeze-thaw cycles, the shear modulus of the samples is 25.67 MPa (C85), 32.16 MPa (C90) and 33.70 MPa (C95), which are 32.6%, 21.4% and 23.1% lower than the samples without freeze-thaw cycles. The ability that resists shear deformation is reduced during the long-term freeze-thaw cycles (> 10).

## Analysis of subsoil-bearing capacity

**Figure 20:**
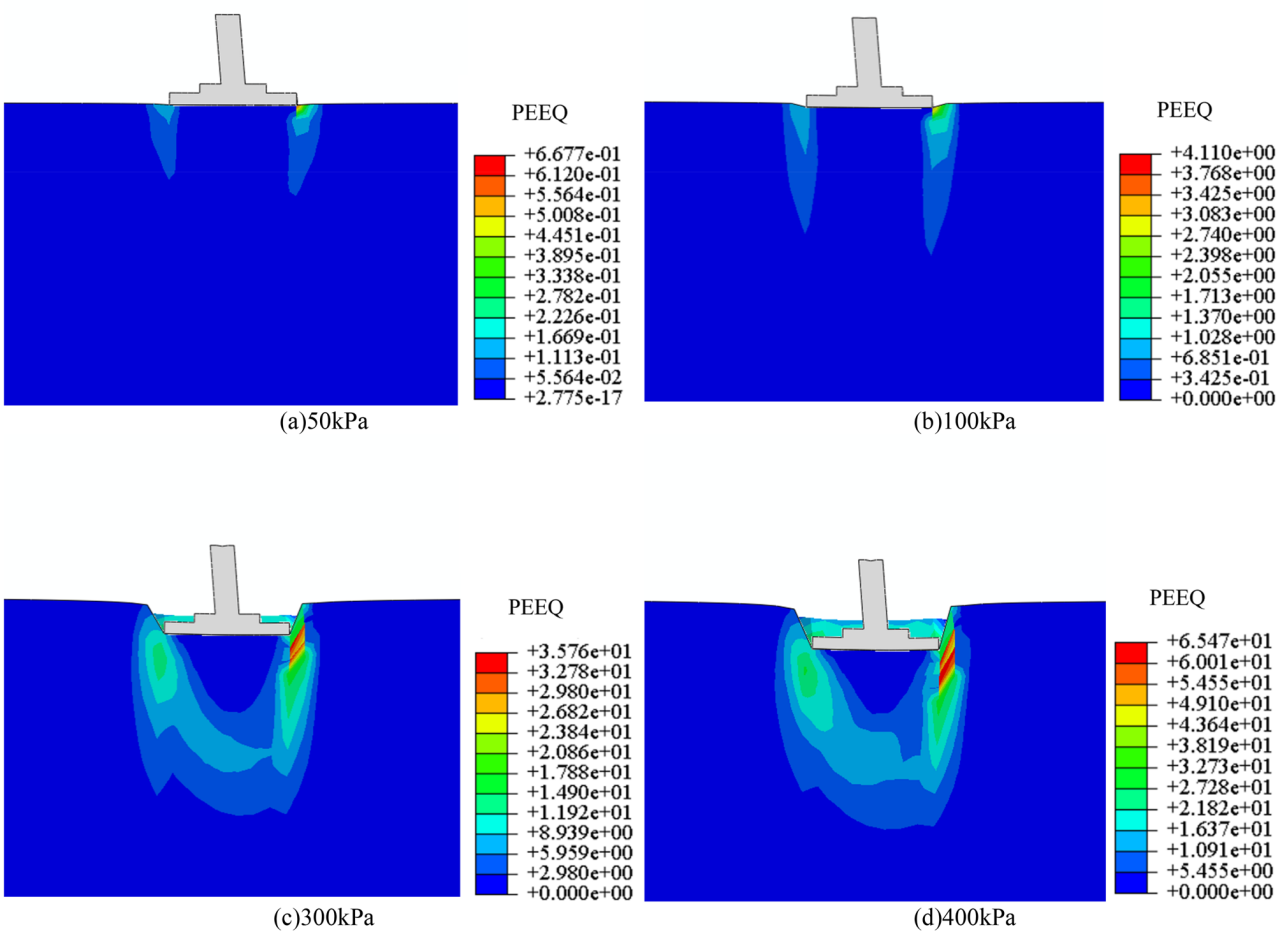
The plastic field of subsoil under different axial loads.

**Figure 21:**
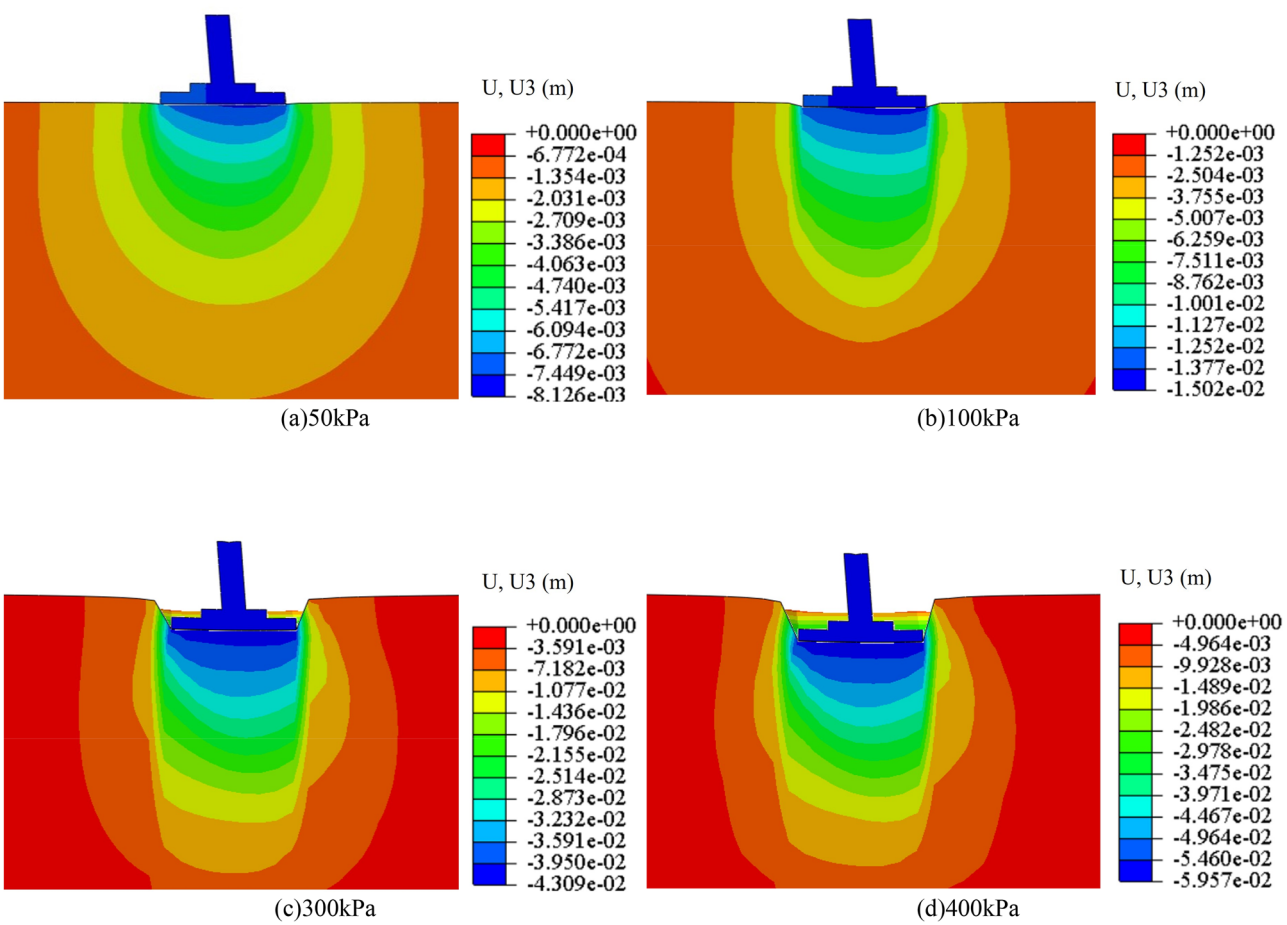
The vertical displacement field of subsoil under different axial loads.

The subsoil-bearing capacity and foundation settlement under different working conditions are calculated by numerical software. Numerical model parameter *N* (*c*, *φ*) is shown in Table 6. The failure form of subsoil depends not only on the type and nature of the soil but also on the characteristics, buried depth and loading conditions of the foundation. Taking the C85 *N* = 0 scenario as an example, an analysis of the variations in the subsoil's plastic zone under different axial loads was conducted, with the computational outcomes illustrated in Fig. 20. With the increase of axial loads, the plastic zone of subsoil develops from the A side to the B side of the foundation, and the range of the plastic zone increases gradually. A plastic zone (The shear failure zone in the subsoil) is penetrated when the pressure to 300 kPa. At this moment, the subsoil is in the stage of a local shear failure. When the to 300 kPa, the plastic zone continues to develop. The vertical displacement of the subsoil is shown in Fig. 21, and the relationship between pressure and settlement is obtained. Because the natural soil has high compressibility, it is difficult to define a real limit value. As pressures mount, even though the subsoil-bearing capacity potential, the deformation of the foundation has surpassed the threshold of normal operational limits; this pressure is thus defined as the ultimate bearing capacity. Therefore, in practical engineering, when the settlement of the foundation is equal to 0.02 times of the width of the foundation (84 mm), the pressure exerted on the top of the foundation is the ultimate bearing capacity of the subsoil. In conclusion, the ultimate bearing capacity of the example is 364 kPa, and the plastic zone of subsoil has been penetrated at the value, so the method for taking the value is reasonable.

**Table 6:** Model parameter *N* (*c*, *φ*).

Cycles (*N*)	*φ* / °	*c* / kPa
0	37.06, 37.26, 44.73 (C85, C90, C95)	15.16, 18.46, 25.79
7	28.17, 34.59, 39.15	20.27, 15.59, 25.79
20	27.71, 29.84, 32.83	8.67, 10.47, 14.96
30	26.56, 26.37, 31.47	4.34, 7.39, 11.76

**Figure 22:**
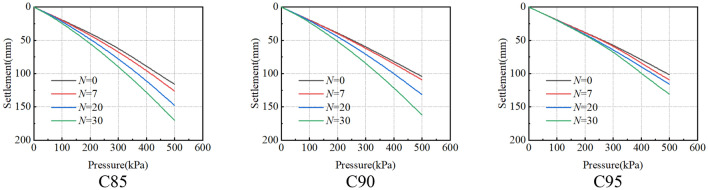
Pressure-settlement curve (p–s curve).

**Figure 23:**
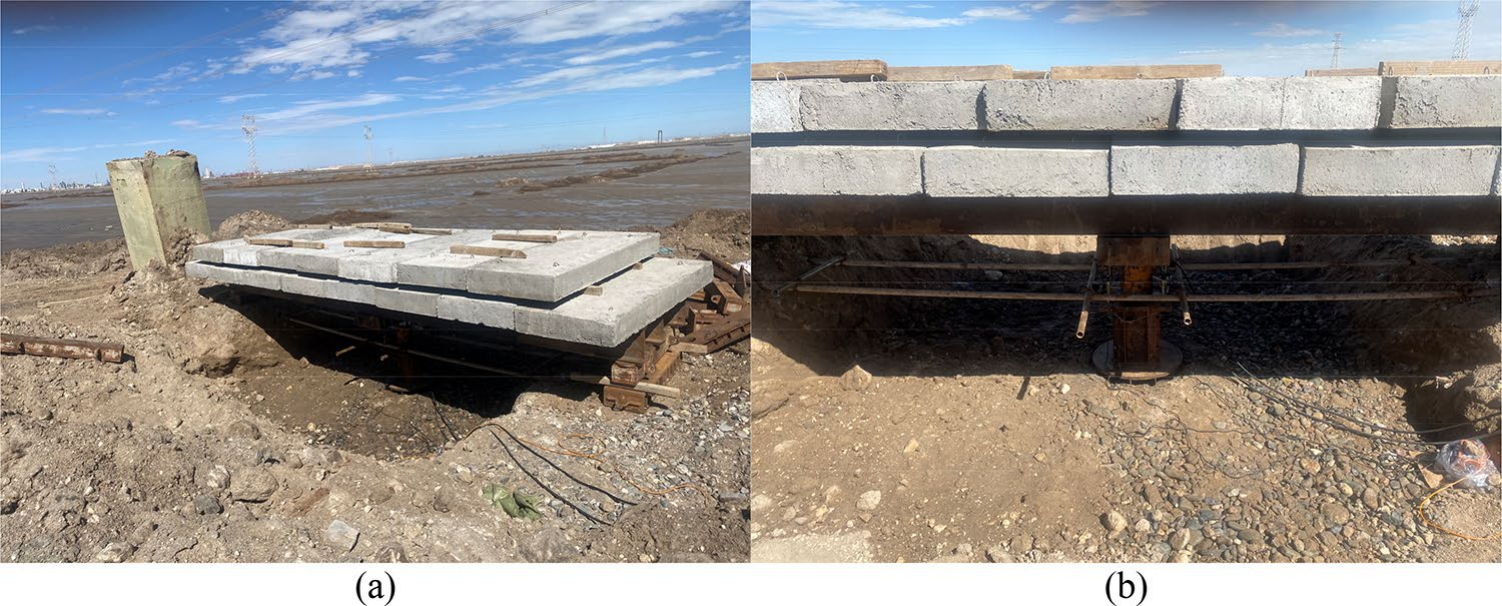
The schematic diagram of in-situ load tests: (**a**) Test device arrangement; (**b**) Apply load to the subsoil.

**Figure 24:**
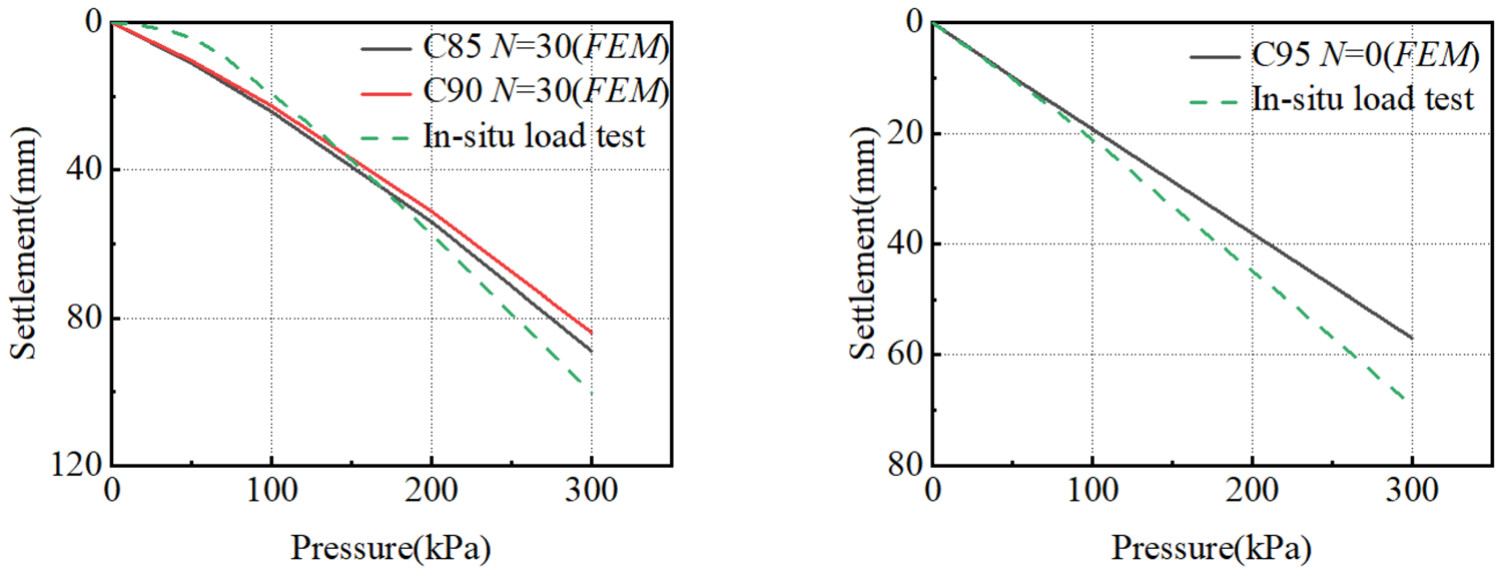
Comparison between the pressure-settlement curve and in-situ load test results: (**a**) Subsoil without compacted compares with C85 and C90; (**b**) Subsoil with compacted compares with C95.

**Figure 25:**
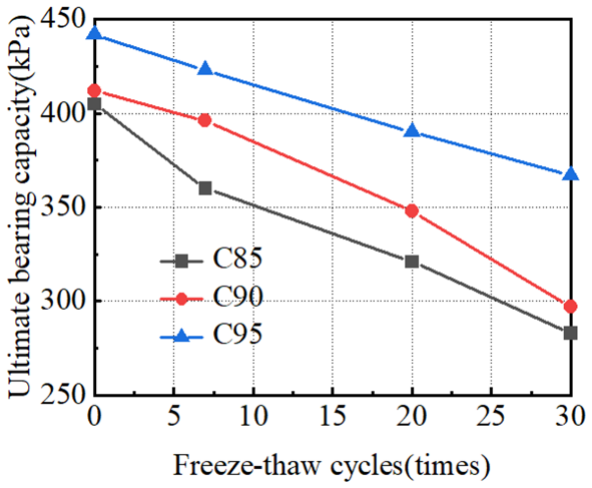
Ultimate bearing capacity of subsoil under different freeze-thaw cycles.

To sum up, the pressure-settlement curves under different working conditions are obtained (Fig. 22). The curve is a downward and slow deformation curve, which has no obvious steep drop, inflexion point and proportional boundary point. The deformation rate of the curve increases with the increase of vertical load, and the difference value of the settlement becomes larger under various loads gradually. According to our previous research in the salt-lake site, in-situ load tests were conducted on both non-compacted (The compactness is between 85 and 90%) and compacted (The compactness is greater than 95%) ground foundations to obtain the p-s curve of an in-situ load test. The numerical model parameters in Table 6 were obtained through the tests. The arrangement for the in-situ load tests is shown in Fig. 23. To verify the rationality of the numerical calculation results in Fig. 22, the numerical calculation results for C85, *N* = 30 and C90, *N* = 30 are compared with In-situ load test results of uncompacted subsoil, and the numerical calculation results for C95, *N* = 0 are compared with the In-situ load test results of compacted subsoil. As shown in Fig. 24, the p-s curve shapes of numerical calculation results are consistent with the in-situ load test results, proving the rational selection of numerical model parameters. Meanwhile, the characteristic value of subsoil-bearing capacity curves under different working conditions is obtained (Fig. 25), and the calculations conform to the test results, which show that the shear strength of soil decreases faster during the long-term freeze-thaw cycle^31^.

As shown in Fig. 25, Ultimate bearing-capacity of subsoil decreases with the increase in freeze-thaw cycles. After 30 freeze-thaw cycles, the ultimate bearing capacity of subsoil is 283 kPa (C85), 297 kPa (C90) and 367 kPa (C95) respectively, which are 30.1%, 27.9% and 16.9% lower than the subsoil without freeze-thaw cycles. Similar to the test results, compactness affects the cementation ability between soil particles and improves the stability of soil structure, C95 has the least reduction in the characteristic value of bearing capacity with freeze-thaw cycles. Therefore, there is an optimum compaction degree between 90 and 95%, which can not only effectively decrease the influence of freeze-thaw action on subsoil, but also decrease the construction cost of tower subsoil in the Salt Lake region. However, after 30 freeze-thaw cycles, the ultimate bearing capacity of subsoil is greater than the design value, so the reduction of subsoil strength caused by freeze-thaw action is not the main reason for the damage of tower structure.

**Figure 26:**
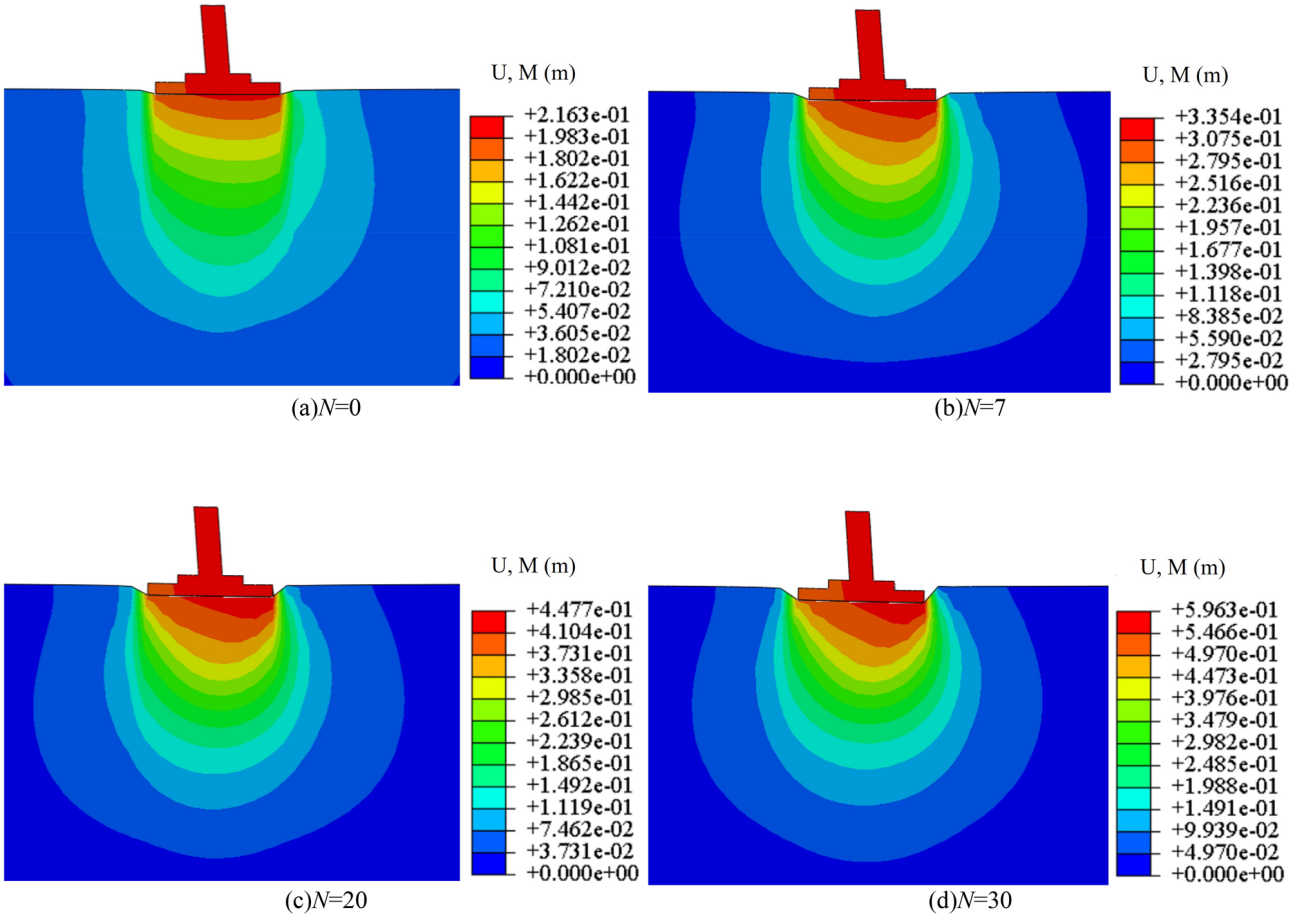
The displacement field of foundation under different axial loads.

**Figure 27:**
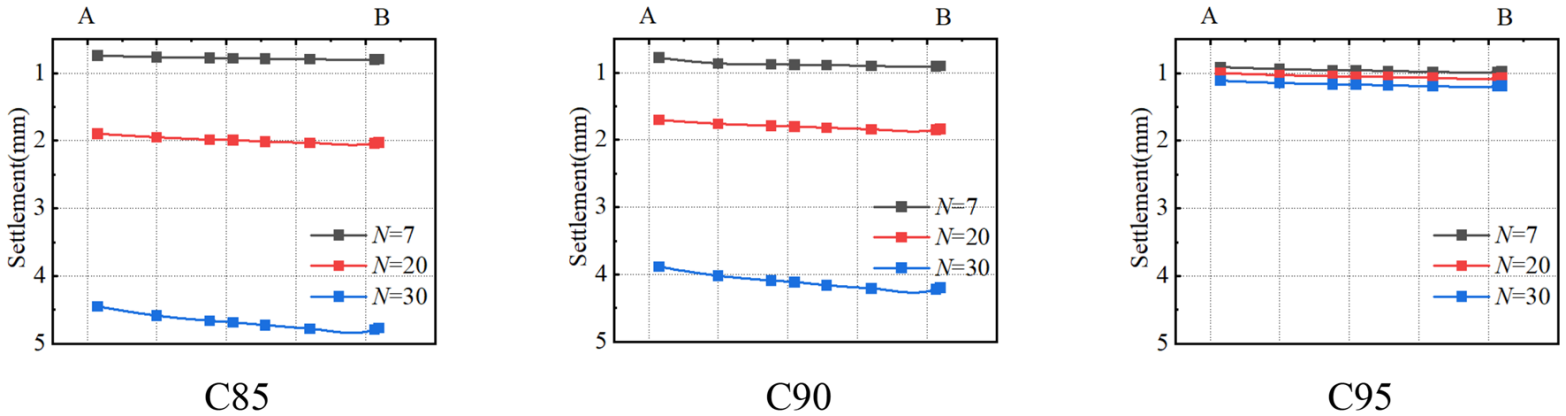
The settlement of the foundation under freeze-thaw cycles.

The deformation of the tower should focus on the settlement at the bottom of the foundation. Taking C85 as an example, when the pressure of 103.2 kPa is applied to the top of the foundation, the settlement of the foundation under different freeze-thaw cycles is shown in Fig. 26. Within the depth of 5 m, the subsoil settlement below the bottom of the foundation gradually decreases from the centre to the edge. The settlement at the bottom of the foundation caused by freeze-thaw action is shown in Fig. 27, which shows the settlement of the bottom of the foundation (A to B) when the load acting on the top of the foundation is an axial load of 1678.5 kN under different freeze-thaw cycles. The figure shows the settlement of the foundation caused by freeze-thaw cycles decreases with the increasing freeze-thaw cycles. After 30 freeze-thaw cycles, the settlement of the foundation caused by freeze-thaw cycles is 4.79 mm (C85), 4.20 mm (C90) and 1.19 mm (C95) respectively. Because the high-density soil shows frost-heaving deformation with freeze-thaw cycles, the foundation settlement of C95 is smaller. Therefore, it is further proved that there is an optimal compaction degree between 90 and 95%, which can reduce the influence of freeze-thaw action on the settlement of the foundation more effectively.

## Discussion

**Figure 28:**
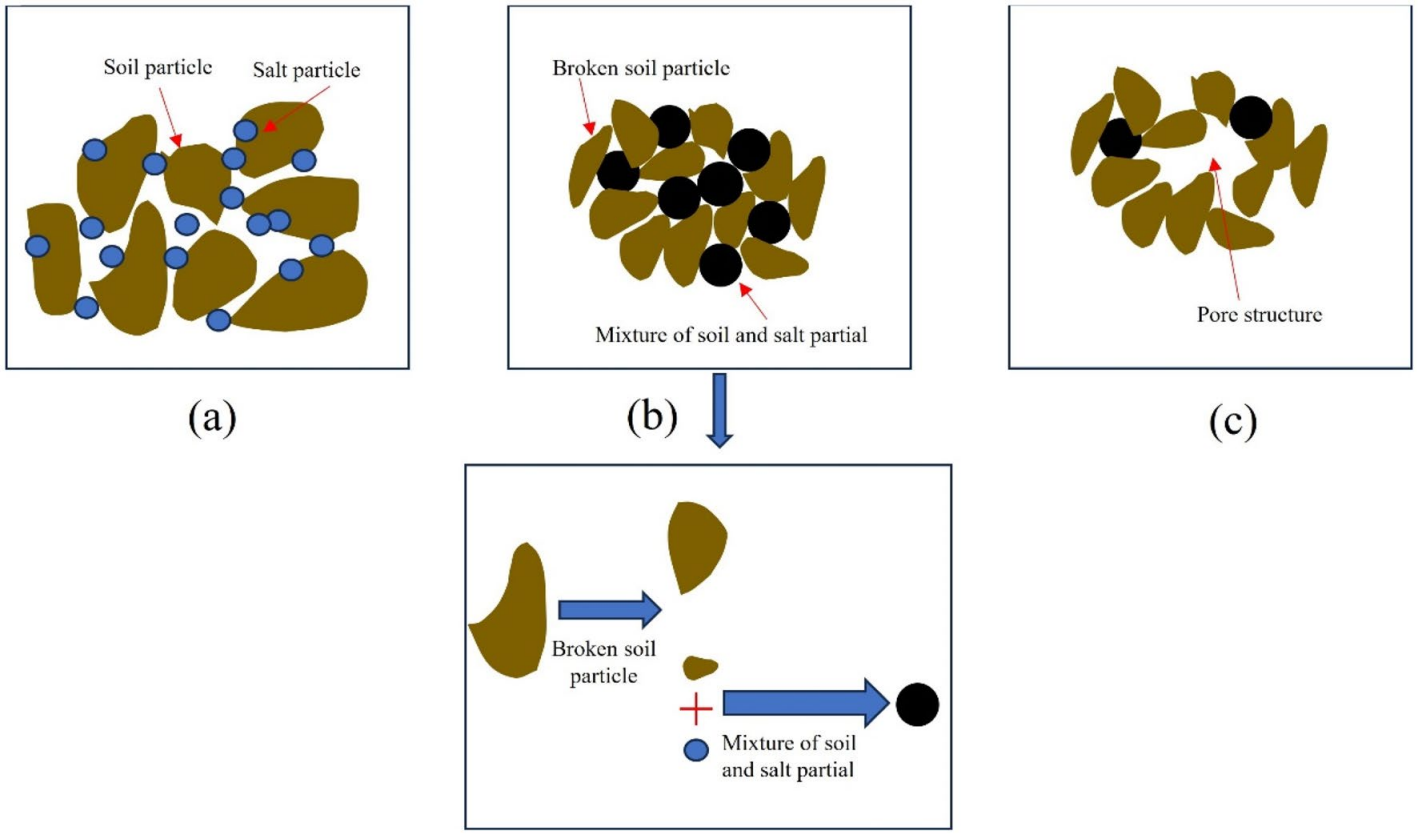
Diagram of soil structure evolution.

According to the numerical calculation results, with the increase in freeze-thaw cycles, the ultimate bearing capacity of subsoil gradually decreases and the settlement of the foundation gradually increases. At the same time, the lower the compaction degree of subsoil, the more significant the settlement of the foundation. The computational outcome stems from the diminution of soil strength caused by freeze-thaw effects. Therefore, in this paper, the strength and deformation evolution of perchlorinated soil under freeze-thaw cycles is analyzed comprehensively from macro and micro aspects. Macroscopically, the cohesion and friction angle gradually decrease during the long-term freeze-thaw cycles, and the low-compaction soil shows thaw settlement deformation. Under the scrutiny of the scanning electron microscope, In the initial state, the soil structure is composed of larger particles^32^, and many salt particles attach to soil particles (Fig. 28a). During the short-term freeze-thaw cycles, the larger soil particles are broken and gradually gather together and form large aggregates under the cementation (Fig. 28b). During the long-term freeze-thaw cycles, a mixture of soil and salt particles is resolved under freeze-thaw action and macropores appear in the soil structure and gradually connect (Fig. 28c). The shear strength parameters of soil samples are related to the changes in the microstructure of the soil. With the increase in freeze-thaw cycles, the total pore area ratio of C90 and C95 gradually increases, and the soil structure tends to become looser, resulting in a gradual decrease in the angle of internal friction. For C85, although the total pore area ratio gradually decreases, there is a noticeable increase in the macropore area ratio, which indicates that the freeze-thaw action has a significant deteriorating effect on the soil structure, which in turn causes a gradual decrease in the friction angle. Besides, Furthermore, for general soil, their cohesion is related to the Coulomb force, van der Waals force, capillary force, and the cementation force between particles. Because the soil samples are strongly saline soil, the soil particles are highly commingled and cemented with the salts, and its cohesion is mainly provided by the inter-particle cementation force. Additionally, the soil-salt cementation forms aggregates, with the interior of these aggregates primarily existing of inter-particle micropores. When the average area of the micropores in the soil samples is small, it indicates a better degree of soil-salt cementation and greater cohesion. Conversely, when the average area of the micropores is large, it shows a poorer degree of soil-salt cementation and weaker cohesion. Therefore, during short-term freeze-thaw cycles, the cohesion of the soil sample gradually increases, while during long-term freeze-thaw cycles, the cohesion of the soil sample gradually decreases.

Besides, the number of macropores in the C85 soil samples is significantly reduced and appears as mesopores, but in the structure of the C90 and C95 soil samples, the originally developed mesopores gradually connect into cracks. Such process would result in the volume of C85 soil samples decreasing gradually, and the volumes of C90 and C95 soil samples increasing gradually. Finally, the cohesion and friction angle of the three soil samples are reduced. Besides, considering that the low-density soil shows thaw settlement deformation and the high-density soil shows frost-heaving deformation under the effect of freeze-thaw cycles, the settlement of the foundation would be reduced, but the amount of reduction is unknown, it would be further improved in the future research^33^.

## Conclusions

In this paper, the natural strongly chlorine saline soil in the Qarhan Salt Lake region was studied. Three soil samples that had different compaction degrees were made and freeze-thaw cycle tests were carried out. The volume, microstructure and shear strength of the soil samples after different freeze-thaw cycles were obtained and discussed. The main results were as follows:

1. With the freeze-thaw cycles, the volume of soil samples in low-density decreases, but the volumes of soil samples in high-density increase. The reason is that the larger soil particles are broken during freeze-thaw cycles. The broken particles gradually gather together, forming large aggregates with the increase of freeze-thaw cycles, and the large aggregates have many cracks. Besides, macropores are reduced significantly in low-density soil samples, but macropores increase significantly in high-density soil samples compared with the soil samples without freeze-thaw cycles.
2. During freeze-thaw cycles, a part of large-size soil particles is decomposed into small-size soil particles leading to the pore structure in soil, and the migration of water in soil also changes the internal structure of soil and redistributing soil particles, resulting in the friction angle decreasing with the increasing freeze-thaw cycles. Besides, forming and breaking soil aggregates is a reason that the cohesion of soil increases during the short-term freeze-thaw cycle (< 7) but decreases during the long-term freeze-thaw cycle (< 10).
3. Enhancing the compaction of soil samples contributes to the reinforcement of soil structure and augments the cohesive capacity After 30 freeze-thaw cycles, the bearing capacity of the loosely compacted subsoil is markedly inferior to that of the densely compacted foundation, with the former experiencing a significantly greater degree of settlement than the latter. Besides, there is an optimum compaction degree between 90 and 95%, which could not only more effectively decrease the influence of freeze-thaw action on subsoil, but also decrease the construction cost of tower subsoil in the Salt Lake region.

## Data Availability

The data used to support the findings of this study are available from the corresponding author upon request.
